# Genome-wide identification, characterization, evolution, and expression pattern analyses of the typical thioredoxin gene family in wheat (*Triticum aestivum* L.)

**DOI:** 10.3389/fpls.2022.1020584

**Published:** 2022-12-22

**Authors:** Jianfei Zhou, Tianqi Song, Hongwei Zhou, Mingfei Zhang, Nan Li, Jishan Xiang, Xiaoke Zhang

**Affiliations:** ^1^ College of Agronomy, Northwest A&F University, Yangling, Shaanxi, China; ^2^ Academy of Agricultural Sciences/Key Laboratory of Agro-Ecological Protection & Exploitation and Utilization of Animal and Plant Resources, ChiFeng University, Chifeng, Inner Mongolia, China

**Keywords:** wheat, typical TRX, gene family, genome-wide, expression pattern

## Abstract

Typical thioredoxin (TRX) plays an important role in maintaining redox balance in plants. However, the typical *TRX* genes in wheat still need to be comprehensively and deeply studied. In this research, a total of 48 typical *TaTRX* genes belonging to eight subtypes were identified *via* a genome-wide search in wheat, and the gene structures, protein conserved motifs, and protein 3D structures of the same subtype were very similar. Evolutionary analysis showed that there are two pairs of tandem duplication genes and 14 clusters of segmental duplication genes in typical *TaTRX* family members; *TaTRX15*, *TaTRX36*, and *TaTRX42* had positive selection compared with the orthologs of their ancestral species; rice and maize have 11 and 13 orthologous typical *TRXs* with wheat, respectively. Gene Ontology (GO) analysis indicated that typical *TaTRXs* were involved in maintaining redox homeostasis in wheat cells. Estimation of ROS content, determination of antioxidant enzyme activity, and gene expression analysis in a line overexpressing one typical *TaTRX* confirmed that *TRX* plays an important role in maintaining redox balance in wheat. A predictive analysis of *cis*-acting elements in the promoter region showed that typical *TaTRXs* were extensively involved in various hormone metabolism and response processes to stress. The results predicted using public databases or verified using RT-qPCR show that typical *TaTRXs* were able to respond to biotic and abiotic stresses, and their expression in wheat was spatiotemporal. A total of 16 wheat proteins belonging to four different families interacting with typical TaTRXs were predicted. The above comprehensive analysis of typical *TaTRX* genes can enrich our understanding of this gene family in wheat and provide valuable insights for further gene function research.

## 1 Introduction

Thioredoxins (TRXs) are ancient, small-molecule proteins that are widely present in prokaryotic and eukaryotic organisms, and mainly act as redox regulators (Holmgren, 1985). TRX forms the TRX system with thioredoxin reductase (TrxR) and the electron-donor nicotinamide adenine dinucleotide phosphate (NADPH) or ferredoxin (Fdx). The system has many functions, such as maintaining redox balance and regulating biological signal transduction (Meyer et al., 2012). TRXs contain the TRX domain in their amino acid sequences, which contain a conserved active site with an amino acid sequence of -Cys-X-X-Cys- (-CXXC-, the X represents amino acids other than Cys). Two cysteine sulfhydryl groups at the active site of a TRX protein can reversibly form disulfide bonds, making TRX existing in two forms: the oxidized state and the reduced state. Disulfide bonds reduced by TRXs are properly formed in the target proteins, thus regulating the activities and spatial structures of the target proteins (Gelhaye et al., 2005). The sequence of the active site of TRX determines the difference in catalytic properties. TRX proteins in plants can be divided into various types according to their active site sequences. TRXs containing the classical active site “WCGPC” are called typical TRXs. Other atypical TRXs include protein disulfide isomerase (PDI) containing the active site of “WCGHC”, chloroplastic drought-induced stress protein32 (CDSP32) containing “HCGPC”, and HCF containing “WCEVC” (Meyer et al., 2005; Chibani et al., 2012). Typical *TRXs* arouse the extensive attention of researchers because of their diverse functions involved in regulating plant growth and development, and alleviating oxidative stress caused by biotic/abiotic stress (Geigenberger et al., 2017).

Typical TRX subtypes in plants include TRX-F, -H, -M, -O, -X, -Y, -Z, and nucleoredoxin (NRX) containing the “WCGPC” active site. These typical TRXs are distributed in the cytoplasm and various organelles of plant cells, and can guarantee the proper progress of various physiological and biochemical activities in plants under normal circumstances by regulating the redox state of various target proteins. Typical TRXs are important regulators of the reactive oxygen species (ROS) scavenging mechanisms and important parts of the antioxidant signal network in plants when they are subjected to oxidative stresses caused by biotic and abiotic constraints (Vieira Dos Santos and Rey, 2006). The TRX-F subtype was originally described as the catalyst for Fructose 1, 6-bisphosphase (FBPase) in spinach (*Spinacia oleracea* L.) (Buchanan, 1980). In *Arabidopsis thaliana*, the adaptability of the *AtTRX-F1* mutant to different light intensities is weakened, and the Calvin–Benson cycle activity and starch accumulation of the *AtTRX-F1* mutant are strongly inhibited, resulting in severe inhibition of the growth of the mutant (Thormählen et al., 2015). The TRX-H subtype receives its name from the word “heterotrophic” because it was first found to exist in non-photosynthetic tissues. Most TRX-H subtypes are localized in the cytoplasm and they have also been found in the endoplasmic reticulum, mitochondria and nucleus (Gelhaye et al., 2004). The expression of the H type thioredoxin gene *AtTRX-H5* in *Arabidopsis thaliana* is closely related to aging, pathogen infection and various kinds of oxidative stress (Christophe et al., 2004). The overexpression of the *NtTRX-H3* gene in tobacco enhances its resistance to paraquat-induced oxidative stress, and its chloroplast damage is lower than that of the wild type. In addition, the overexpression of the *NtTRX-H3* gene in tobacco enhances its resistance to tobacco mosaic virus (TMV) and cucumber mosaic virus (CMV) (Sun et al., 2010). TaTRX-H1 interacts with TaCP1 (a RD19-like cysteine protease) and participates in the regulation of the salicylic acid (SA) related defense signaling pathway, thus regulating wheat resistance to stripe rust (Shi et al., 2021). Multiple ABA response *cis*-elements have been predicted in the 1500 bp region upstream of the start codon of the *TaTRX-h9* gene, and the expression of *TaTRX-H9* in wheat leaves is induced by drought stress and ABA (Li et al., 2019). TRX-M proteins were initially described as catalysts for NADPH-dependent NADP-malate dehydrogenase (NADP-MDH) (Buchanan et al., 1980). *TRX–M*–mediated redox regulation has important effects on leaf structure, gas exchange, energy transfer, and anthocyanin accumulation. Based on the study of *AtTRX-M* depletion mutants in *Arabidopsis*, it was found that AtTRX-M protein plays an active role in the resistance of Arabidopsis to high light intensity stress (Serrato et al., 2021). The knockout of *OsTRX-M* in rice results in abnormal chloroplast development and inhibits the growth of the plants (Chi et al., 2008). O-type TRX has a predicted N-terminal mitochondrial target peptide, which was subsequently confirmed by subcellular localization in the mitochondria (Laloi et al., 2001). AtTRX-O1 was found to regulate the thiol redox status of succinate dehydrogenase and fumarase in the tricarboxylic acid (TCA) cycle, so as to regulate the activities of these two enzymes and ensure the smooth operation of the TCA cycle (Daloso et al., 2015). After treatment with a high concentration of H_2_O_2_, the overexpression of *PsTRX-O1* (*Pisum sativum* L.) in tobacco is associated with increased catalase activity in cells and reduced content of endogenous H_2_O_2_, and finally keep the cell vitality of the tobacco overexpressing the *TRX-O1* gene unchanged or make it even higher (Ortiz-Espín et al., 2015). *TRX-X* was originally identified in the genome of *Arabidopsis thaliana*, and its encoded protein exists in the chloroplast. The determination of kinetic parameters shows that TRX-X is a very effective reducing agent for 2-cys peroxiredoxin (Prx) (Collin et al., 2003). TRX-Y has also been identified in the chloroplast of Arabidopsis, and the *TRX-Y* gene is mainly expressed in leaves and induced by light (Collin et al., 2004). *TRX-Z* has high homology with *TRX-X* and *TRX-Y* based on phylogenetic analysis. Functional loss analyses of TRX-Z in *Arabidopsis thaliana* and *Nicotiana bentosa* have shown that TRX-Z interacts with two fructokinase-like proteins (FLN) to regulate chloroplast development (Arsova et al., 2010). Laughner et al. (1998) identified a new type of TRX in the nucleus from maize (*Zea mays* L.) and named it nucleoredoxin (NRX). NRX in plants can interact directly with phosphofructokinase 1 (PFK1) to regulate PFK1 activity, thus maintaining a balance between the glycolysis and pentose phosphate pathways (Funato et al., 2013). The overexpression of *TaNRX1* (*TaTRX24* in this study) in wheat can lead to an increase in gene expression related to hormone signal transduction, phenylpropane biosynthesis, carbon metabolism, and nitrogen metabolism, thus significantly enhancing the drought resistance of wheat (Zhang Y. R. et al., 2021). The aforementioned results indicate that typical TRXs in plants can affect and regulate a variety of metabolic pathways and improve defense against adverse stresses in plants due to the diversity of family members and their extensive distributions in cells.

Wheat is one of the most important crops globally and is widely grown in different geographical, climatic and ecological regions around the world (Yue et al., 2019). The world’s wheat production in 2020 was about 760.9 million tons, as counted by the Food and Agriculture Organization of the United Nations (FAO) (https://www.fao.org/faostat/-en/#data/QCL/visualize). At present, the challenges of global climate change and population surge are becoming increasingly serious. The stability of wheat yields is the top priority to ensure world food security and economic development (Pankaj et al., 2022). The yield and quality of wheat are severely affected by biotic and abiotic stresses such as disease, drought, high temperature, cold, high salinity and so on. In order to cope with and adapt to these stresses, wheat has evolved a complex regulatory network, which can regulate different biochemical and physiological processes under stress conditions (Kumar et al., 2022; Wang et al., 2022a; Wang et al., 2022b). Therefore, it is necessary to improve the stress resistance of wheat, reduce the influence of stress on the growth and development of wheat, and ultimately improve the yield and quality of wheat by exploring and studying the genes related to stress regulation in wheat and selecting and utilizing them in the breeding process. Wheat is a heterologous hexaploid with a large and complex genome consisting of three subgenomes (A, B, and D), making it an ideal model for studying polyploidy and the subfunctionalization of plant homologous genes. The completion of high-quality reference sequences enables researchers to study the gene distribution and evolutionary dynamics of wheat gene families at the genome-wide level (IWGSC, 2018). Bhurta et al. (2022) identified 42 *TRX* genes in wheat, and then selected 15 of them based on *in silico* expression analysis for bioinformatics comprehensive analysis, and further studies related to stripe rust showed that four *TaTRX* genes were significantly induced in the response to stripe rust infection, and that *TRX* can maintain the ROS homeostasis of wheat cells between 24h and 72h after wheat leaves were inoculated with stripe rust. However, all of the typical *TRX* genes in wheat have not yet been comprehensively and systematically studied. In this study, we performed a genome-wide search and identification of the typical *TRX* gene family in wheat, and then systematically analyzed the chromosomal location, phylogenetic relationship, gene structure, protein conserved domain, protein 3D structure, gene ontology, the relationship between redox status and *TRX* gene expression, *cis*-acting elements, and protein interaction networks. We also analyzed the spatiotemporal expression characteristics of typical *TaTRX* genes and the expression characteristics of typical *TaTRX* genes under abiotic or biotic stresses such as drought, high temperature, low temperature, salt, stripe rust and powdery mildew. The results obtained extend our understanding of the biological functions and evolutionary history of typical *TaTRX* genes and their encoded proteins in wheat, lay a foundation for further studies on the functions of typical *TRX* gene families in wheat and other crops, and enable us to provide candidate genes for breeding wheat varieties with strong stress resistance.

## 2 Materials and methods

### 2.1 Identification and characterization analyses of typical *TaTRX* genes

The coding DNA sequence (CDS), protein sequence and general feature format version 3 (GFF3) files of *Triticum aestivum* L. were downloaded from the Ensembl Plants database (http://plants.ensembl.org/Triticum_aestivum/Info/Index/). The hidden Markov model (HMM) of TRX (PF00085) was downloaded from the Pfam database (http://pfam.xfam.org/). The HMMER 3.1 search tool was used to screen the wheat TRX proteins. The cutoff value for the HMMsearch program was 0.001. The putative TaTRXs were subsequently submitted to the NCBI-CDD server (https://www.ncbi.nlm.nih.gov/cdd/), SMART database (http://smart.embl.de/) and HMMscan (https://www.ebi.ac.uk/Tools/hmmer/search/hmmscan/) to confirm the existence of the TRX domain. Finally, proteins containing the “WCGPC” sequence in the TRX domain were screened out, and were named according to their chromosomal locations. The same method was used to identify the typical *TRX* genes in *Triticum turgidum* (emmer wheat), *Aegilops tauschii*, *Arabidopsis thaliana*, *Oryza sativa* (rice), and *Zea mays* (maize). The chromosome locations of the typical *TaTRX* genes were obtained from the GFF3 files and were then visualized using the Mapchart (v2.32) software. The molecular weight (Mw), isoelectric point (pI), and grand average of hydropathicity (GRAVY) values of these identified proteins were investigated using ExPASy online tools (http://web.expasy.org/protparam/). Their subcellular localizations were predicted based on the CELLO online tool (v2.5, http://cello.life.nctu.edu.tw/) and the WoLF PSORT online tool (https://www.genscript.com/wolf-psort.html/).

### 2.2 Gene structures and conserved motifs of typical *TaTRX* genes and TaTRX proteins

The exon–intron organizations and untranslated regions (UTRs) of these predicted typical *TaTRX* genes were retrieved from the GFF3 files of the wheat genomes using the BMKCloud online tool (http://www.biocloud.net/), then they were graphically displayed using the Gene Structure Display Server (GSDS) online tool (v2.0, http://gsds.gao-lab.org/). Conserved motifs were predicted and displayed using the MEME online tool (v5.4.1, http://meme-suite.org/), and the parameters were set as follows: the maximum number of each motif was set at 10 and the optimum width of each motif was set to between 5 and 200 residues.

### 2.3 Predictions of typical TaTRX 3D structures

Proteins with similar amino acid sequences usually have similar three–dimensional (3D) structures, therefore, the successfully resolved 3D structure of the protein with a consistency of ≥ 30% with the amino acid sequence of the target protein was used as the template. 3D structures of typical TaTRX proteins can be predicted and drawn using the Swiss-Model interactive tool (https://swissmodel.expasy.org/) according to the homology modeling method. A template with a high degree of coverage and identity (≥ 30%) was selected preferentially using the Swiss-Model tool. Then, we selected the template with a global model quality estimation (GMQE) score of close to 1 and a QMEAN score of close to 0 from the above screening results. Finally, a Ramachandran plot was used to evaluate the quality of the 3D model constructed above.

### 2.4 Phylogenetic and collinearity analyses of typical *TaTRX* genes

To explore the evolutionary relationships of the typical *TaTRX* genes, multiple sequence alignments of the identified typical TRX proteins of wheat, Arabidopsis, rice and maize were performed using the ClustalW tool, and an evolutionary tree was constructed using the neighbor-joining (NJ) method with MGEA 8.0 software, with the bootstrap value set to 1000. Then, the Evolview online tool (v2.0, https://evolgenius.info//-evolview-v2/#login/) was used to refine the NJ–tree constructed above.

The MCScanX tool was used to study gene duplication and collinearity. The Ka (non-synonymous substitution)/Ks (synonymous substitution) values between common wheat and *Triticum turgidum*/*Aegilops tauschii* were calculated to assess the molecular selection effect using the KaKs-Calculator tool. The genes with Ks values of more than 0.3, as well as Ka and Ks values of 0, were discarded, because they might result from sequence saturation or misalignment (Guo et al., 2017).

### 2.5 GO enrichment analyses of typical *TaTRX* genes

The typical *TaTRX* genes were functionally annotated using GO terms in three main categories (CC, BP, and MF) with the TGT online tool (http://wheat.cau.edu.cn/TGT/). The Bonferroni correction method was used to adjust the *p*-values. The number of genes in the background ranged from 5 to 1200, and 0.05 was applied as the false discovery rate for the filter.

### 2.6 *Cis*-regulatory element analyses

The 2,000 bp genome sequences upstream of the typical *TRX* genes transcription initiation sites in wheat were downloaded from the Ensembl Plants database (http://plants.ensembl.org/Triticum_aestivum/Info/Index/) to further identify the putative *cis*-regulatory elements in the promoter regions of the typical *TaTRX* genes. The various putative *cis*-regulatory elements in the above sequences were predicted using the PlantCARE online tool (http://bioinformatics.psb.ugent.be/webtools/plantcare/html/), and then graphically displayed using the GSDS online tool (v2.0, http://gsds.gao-lab.org/) and TBtools software (v0.664432).

### 2.7 Protein interaction network analyses of typical TaTRXs

In order to identify other wheat proteins that interact with typical TaTRXs, the protein interaction network involving typical TaTRXs was studied using the STRING online tool (v11.5, http://string-db.org/cgi/) based on the protein interaction networks in Arabidopsis and the orthologous genes between wheat and Arabidopsis. The predicted interaction network was displayed using the Cytoscape software (v3.8.2).

### 2.8 Expression analyses of the typical *TaTRX* gene family from RNA-Seq data

To study the expression profiles of typical *TaTRXs*, a total of 87 publicly available RNA-seq samples from five tissues (root, stem, leaf, spike, and grain) and six biotic/abiotic stress conditions (drought, heat, salt, cold, powdery mildew and stripe rust) were downloaded from Wheat URGI (http://www.wheat-expression.com/) and the NCBI Sequence Read Archive (SRA) database (https://www.ncbi.nlm.nih.gov/). The accession numbers and sample information are listed in **Table S6**. Subsequently, the expression levels of typical *TaTRX* genes were calculated and normalized with TPM (transcripts per million) values, and heatmaps were generated using the pheatmap package in R software.

### 2.9 Plant materials and stress treatments

In order to verify the accuracy of transcriptome data in the database, the wheat variety Chinese Spring (CS) was cultivated and grown in a climate chamber (70% humidity, 18,000 Lux light intensity) with 25°C/20°C (day/night), 16 h/8 h (light/dark). The concentration of the Hoagland solution used in this study was 50% of the standard. Wheat seedlings at the two-leaf-stage were subjected to 20% PEG 6000 (dehydration stress), 37°C (heat stress), 4°C (cold stress), and 200 mmol/L NaCl (salt stress) as stress treatments. Wheat leaves were collected after 0, 1, 2, 6, 12, and 24h, and the leaves treated at 0h served as controls for the stress treatments. All fresh samples were quickly frozen in liquid nitrogen and stored in an ultra-low temperature freezer (-80°C).

To investigate the relationship between the redox status and *TRX* gene expression, the seeds of two contrasting wheat genotypes, JW (selected from a segregated population of the cross of spring common wheat cultivars “Fielder” and “NB1”) and the *TaNRX1* (*TaTRX24* in this study) –overexpression (OE) line, a transgenic wheat line overexpressing the *TaTRX24* gene in JW background (Zhang Y. R. et al., 2021), were used for the current experiment. JW was used as a wild–type (WT) receptor material in this study. The germinated seeds of the WT and OE wheat lines were cultivated with a 50% Hoagland nutrient solution. Plants were cultivated in a light incubator at 20-25°C, with a 16 h light/8 h dark photoperiod, humidity at approximately 70%, and light intensity at 18,000 Lux. When the wheat grew to the stage of two leaves and one heart, the nutrient solution was replaced by a 50% Hoagland nutrient solution with 20% (M/V) polyethylene glycol (PEG 6000). Sampling was conducted by collecting the leaves at 0 HAD (hours after dehydration stress treatment), 24 HAD, 48 HAD, 72 HAD, and 96 HAD for analysis.

### 2.10 Measurement of the relevant physiological indicators

Superoxide dismutase (SOD), peroxidase (POD), and catalase (CAT) contents were measured using a SOD, CAT, and POD test box (Suzhou Keming Biotechnology Co., Ltd, Suzhou, China). The H_2_O_2_ content was measured using a H_2_O_2_ test box (Suzhou Keming Biotechnology Co., Ltd, Suzhou, China). The O_2_^-^ level was measured according to the method described by Wang et al. (2017). Malondialdehyde (MDA) content was measured using the thiobarbituric acid method (Zhang et al., 2020).

### 2.11 RNA extraction and RT-qPCR

The total RNA was extracted using the TRIzol method (TIANGEN, China). The cDNA was synthesized with a reverse transcription kit (TransGen Biotech, China). The details of all the specific primers are listed in **Table S7**. Real–time quantitative PCR (RT-qPCR) was performed using the ABI life fluorescence quantitative PCR System Q3. Reactions of 20 µL contained 2×*PerfectStart* ^®^ Green qPCR SuperMix 10 µL, Passive Reference Dye (50×) 0.4 µL, 0.4 µL forward and reverse primers for each (0.2 μM), 1 µL cDNA (200 ng/μL), and 7.8 µL nuclease–free water. The reaction conditions applied were 94°C for 30s, 40 cycles of 94°C for 5s, and 60°C for 30s.

### 2.12 Statistical analysis

All experiments were independently repeated at least three times to obtain reliable data for statistical analysis. Statistical analysis was performed using SPSS 22.0 (SPSS, Chicago, Illinois, USA). The *parametric test* one-way analysis of variance was used for data analysis, and the least significant difference *post hoc* test was used to determine significant differences between means (*, statistically significant at *P* < 0.05; **, statistically significant at *P* < 0.01). The transcript abundance levels were calculated using the 2^–ΔΔCt^ method with the wheat *β-actin* gene as the internal control.

## 3 Results

### 3.1 Identification of the typical *TRX* gene family in wheat

**Figure 1:**
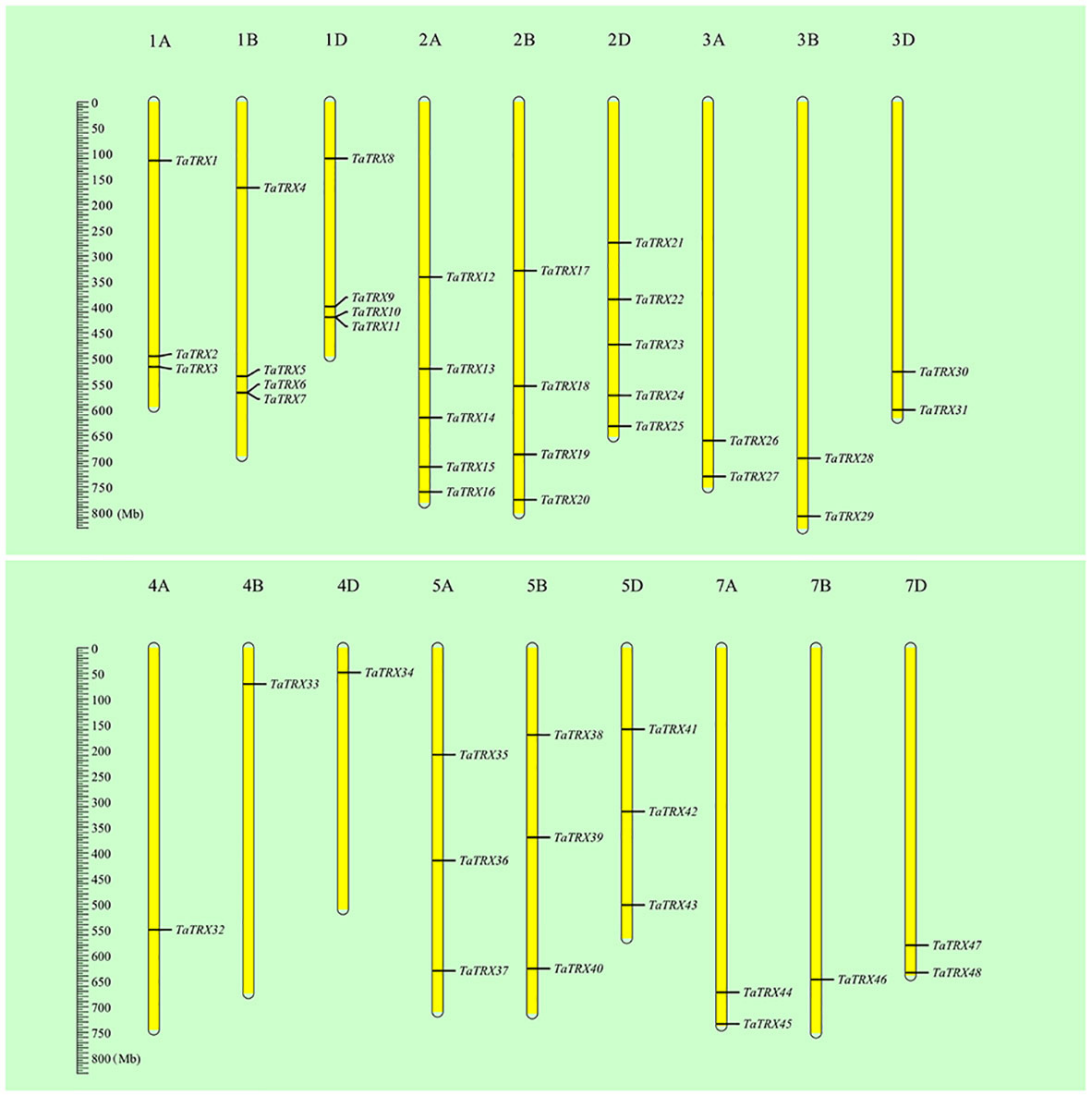
Relative positions of typical *TaTRX* family members in wheat on chromosomes. All wheat chromosomes were drawn to scale based on their actual physical lengths.

In order to identify the typical members of the *TaTRX* gene family in wheat, the hidden Markov model (HMM) profile of the TRX domain (PF00085) was retrieved from the wheat protein database (IWGSC RefSeq v1.0) using the HMMsearch program (HMMER3.1 package), and then confirmed using the SMART database, HMMscan, and the NCBI–CDD server. In the retrieved TaTRX protein sequences, proteins containing the “WCGPC” sequence in the TRX domain were screened, and 48 typical *TaTRX* gene family members were finally identified. They were named from *TaTRX1* to *TaTRX48*, ordered according to their locations in chromosomes from 1A to 7D, and from top to bottom (**Figure 1**).

The basic information in respect of the typical *TaTRX* family members identified above is listed in **Table S1**. The proteins of this family members are composed of at least 95 amino acids (TaTRX17), and at most 525 amino acids (TaTRX24). The molecular weights of this family of proteins ranged from 10.39 kDa (TaTRX17) to 57.96 kDa (TaTRX15). The isoelectric points (pIs) ranged between 4.47 (TaTRX5) and 9.77 (TaTRX22), of which 22 family members had pIs greater than 7, and 26 family members had pIs less than 7. The grand average of hydropathicity (GRAVY) values of all family members were between -0.543 and 0.442, of which 14 family members had GRAVY values greater than 0, indicating that these proteins were hydrophobic, while 34 family members had GRAVY values that were less than 0, indicating that these proteins were hydrophilic. Chromosome mapping results (**Figure 1**) showed that typical *TaTRX* family members were distributed on 18 chromosomes except for 6A, 6B and 6D. Chromosomes 2A and 2D contained the maximum number of *TaTRXs*, while 4A, 4B, 4D and 7B had the minimum number of *TaTRXs*. In order to obtain information for the study of family member functions, predictions of subcellular locations were carried out (**Table S1** and **Table S2**). The results show that a total of 23 typical TaTRX family members were localized in the cytoplasm (H type and NRX type), among which TaTRX2 was localized in both the cytoplasm and extracellular matrix, and TaTRX15, TaTRX19 and TaTRX24 were all localized in the cytoplasm and nucleus. In addition, 22 members were predicted to be localized in the chloroplast (F type, M type, X type, Y type, and Z type), and three members were predicted to be localized in the mitochondria (O type). The diversity of subcellular localization meant that typical TaTRXs might be widely involved in a variety of biochemical processes in wheat cells.

### 3.2 Phylogenetic relationship, conserved motif, gene structure and protein homology modeling analyses

**Figure 2:**
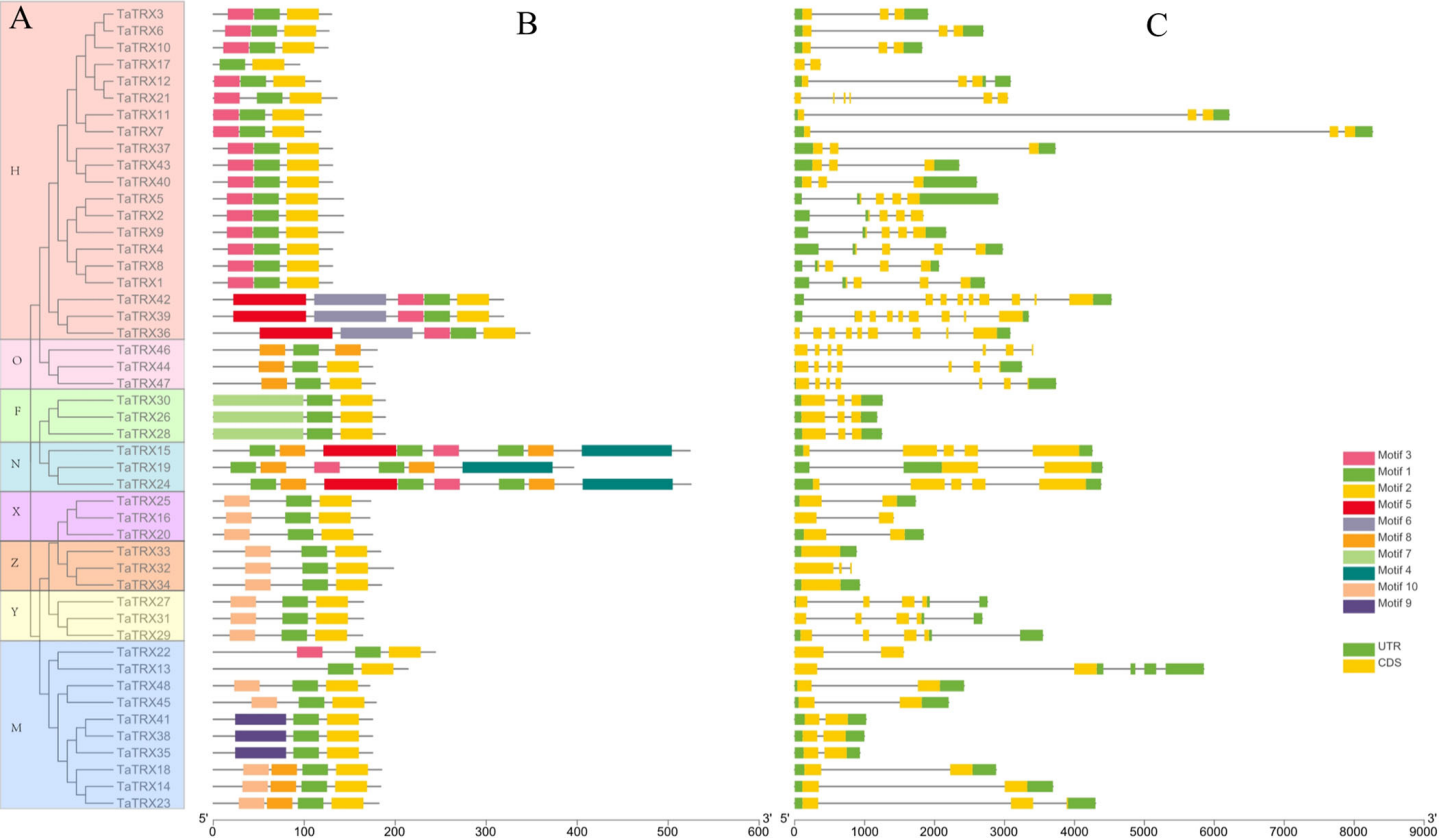
Phylogenetic relationships **(A)**, conserved motifs **(B)**, and gene structures **(C)** of typical *TaTRX* genes in wheat. The different colors in A represent the 8 subtypes of typical *TaTRXs*. The different colors in B represent the 10 identified motifs. The yellow and green rectangles in C represent the coding sequences (CDSs) and untranslated regions (UTRs), respectively, and the black lines represent introns. The length of the CDS, UTR, and intron for each typical *TaTRX* gene was shown proportionally.

A total of 17 homoeologous groups were identified *via* multiple sequence alignment and cluster analysis of the protein sequences of typical TaTRXs in wheat. Combined with their respective amino acid sequence features and annotation information in the Ensembl Plants database (http://plants.ensembl.org/Triticum_aestivum/Info/Index), the identified 48 typical *TRX* genes were divided into eight subtypes: F, H, M, O, X, Y, Z, and NRX (**Figure 2A**). Among them, the H type comprised the largest number of *TRX* genes (20), followed by the M Type (10). A typical *TaTRX* of the other six subtypes contained three homologous genes each.

Analyses of protein conserved motifs and gene structures can provide important and valuable information for the study of gene function diversification. The protein sequences of typical TaTRXs were submitted to the MEME website, and a total of 10 conserved motifs were identified (**Figure 2B**, **Figure S1**, and **Table S3**). Motif 1, which contains the “WCGPC” sequence, was located in the TRX domain, and all typical TaTRX proteins contained this motif (**Figure S2**). All TRX-H members contained motif 1, motif 2, and motif 3; all TRX-X, TRX-Y, and TRX-Z members contained motif 1, motif 2, and motif 10; and only TRX-F members contained motif 7. Most of the proteins of typical TaTRXs contained three conserved motifs, and motifs in same subtype of TaTRXs were very similar.

The results of gene structure analyses showed that *TaTRX7* had the longest gene length and intron length, while *TaTRX17* had the shortest gene length (**Figure 2C**). The numbers of introns ranged from 0 to 8, and the numbers of exons varied from 1 to 9. Only Z-type *TaTRX33* and *TaTRX34* had no introns among all family members, while *TaTRX36*, *TaTRX39* and *TaTRX42* contained more exons than other family members. In general, the gene structures of genes with a close genetic relationship were also more similar.

The 3D structures of these typical TaTRX proteins were predicted using homology-based modeling (**Figures S3A–E**). The predicted 3D models of 45 proteins had global model quality estimation (GMQE) values of greater than 0.8 and QMEAN values that were close to 0, and residues up to 90% fell in the most favored regions in the Ramachandran plot (**Figures S4A–E**), which show that these predicted 3D models were relatively reliable. It can be seen from the above prediction results that typical TaTRX proteins may be composed of four peripheral *α*-helices, and five anti-parallel and cross-stacked *β*-sheets inside. The second *α*-helix and the second *β*-sheet were connected by the active site “WCGPC”, which is very similar to the 3D structural model of *Escherichia* *coli* (*E. coli*) TRX (Martin, 1995).

### 3.3 Tandem duplication and genome collinearity analyses of typical *TaTRX*s

**Figure 3:**
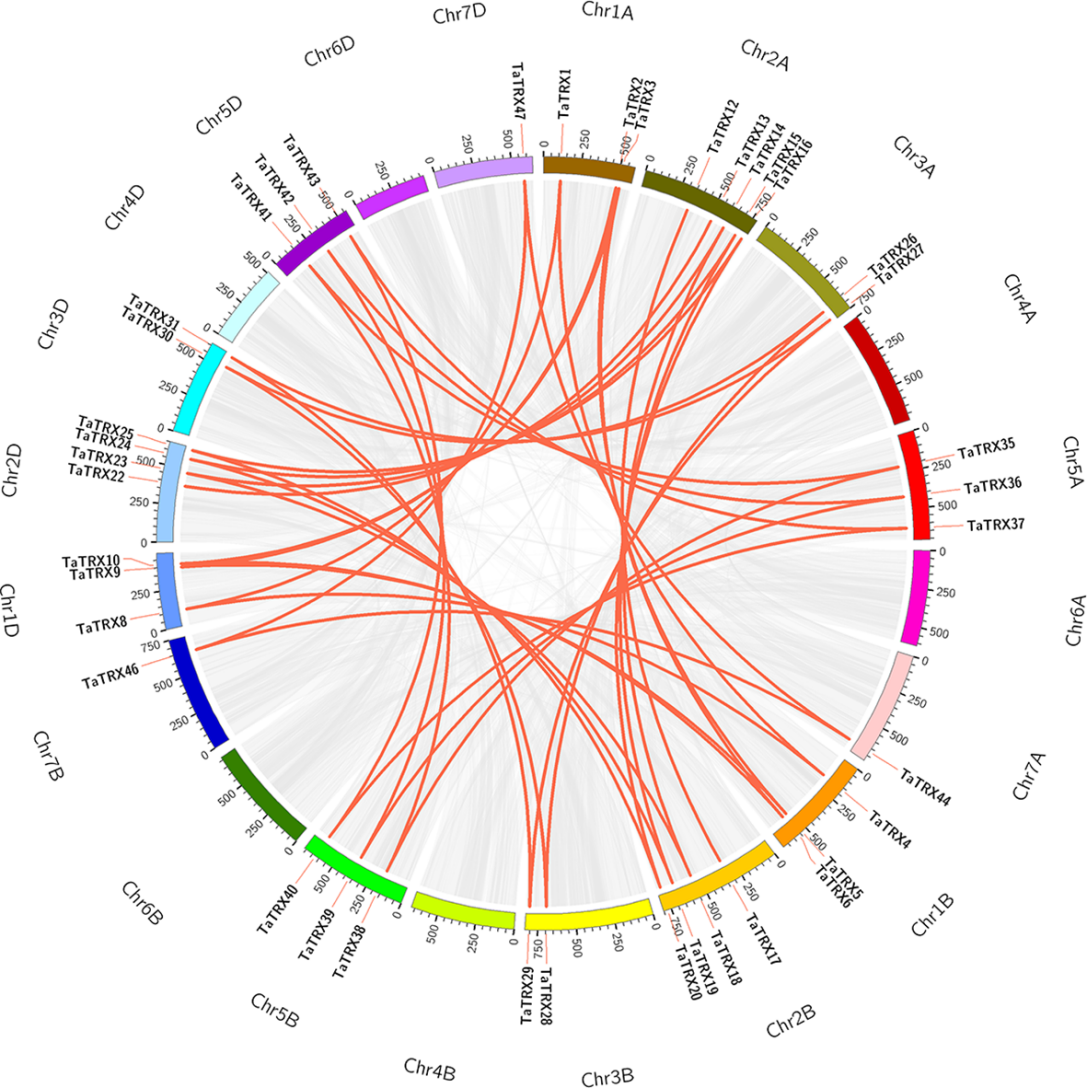
Distribution and duplication events of typical *TaTRX* genes across wheat genome. All typical *TaTRX* genes were mapped to 21 wheat chromosomes in a circle using Circos tool, and segmental duplications were mapped to their respective locations. Gray regions indicated all synteny blocks within the wheat genome, while red lines represented segmental duplications. The chromosome numbers were marked outside of the circle.

According to the analysis results of the MCScanX program package and the chromosomal position information of typical *TaTRXs* (**Figure 1**, **Table S1**), two pairs of tandem duplication genes were screened out, namely *TaTRX6* and *TaTRX7*, and *TaTRX10* and *TaTRX11*. Their relative distances on chromosomes were 135.6Kb and 243.5Kb, respectively. Collinearity analyses of the MCScanX program package showed that there were 14 clusters of segmental duplication genes in the typical *TRX* family of wheat. Among the homologous gene clusters of *TaTRX12*, *TaTRX17*, and *TaTRX21*, only *TaTRX12* and *TaTRX17* exhibited the phenomenon of segmental duplication (**Figure 3**). The above results suggest that tandem duplication and segmental duplication might promote the expansion of members of the typical *TaTRX* gene family.

### 3.4 Phylogenetic analyses and collinearity relationships of typical *TRX* genes among different plants

To analyze and evaluate the evolutionary relationships of typical *TaTRXs*, typical TRX protein sequences of wheat, Arabidopsis, rice, and maize were compared, and phylogenetic trees were constructed (**Figure S5**, **Table S4**). As shown in **Figure S5**, 20, 17, and 20 typical *TRX* genes were identified in Arabidopsis, rice, and maize, respectively, and the typical *TRXs* of the same subtypes were grouped together with their orthologous counterparts.

The collinearity of typical *TRX* gene families of these important crops was analyzed by constructing collinearity maps of wheat (*Triticum aestivum* L.), rice (*Oryza sativa* L.), and maize (*Zea mays* L.) (**Figure S6**). The results show that 11 genes in rice had orthologous relationships with 26 typical *TRX* genes in wheat, and 13 genes in maize had orthologous relationships with 19 typical *TRX* genes in wheat. Specifically, the typical *TaTRX1/4/8* genes may have a common genetic origin with the typical *OsTRX1/10* genes and the typical *ZmTRX7/13* genes; the typical *TaTRX13/22* genes may have a common genetic origin with the typical *OsTRX7* gene and the typical *ZmTRX5* gene; the typical *TaTRX14/18/23* genes may have a common genetic origin with the typical *OsTRX4/8* genes and the typical *ZmTRX4/10/12* genes; the typical *TaTRX16/20/25* genes may have a common genetic origin with the typical *OsTRX9* gene and the typical *ZmTRX3/20* genes; the typical *TaTRX29* gene may have a common genetic origin with the typical *OsTRX3* gene and the typical *ZmTRX9* gene; and the typical *TaTRX37/40/43* genes may have a common genetic origin with the typical *OsTRX6* gene and the typical *ZmTRX2/11* genes. The above results will assist in the study of the evolutionary history and even the gene function of the typical *TRX* gene family in wheat, rice, and maize.

### 3.5 Molecular evolution analyses of typical *TRX* genes in wheat and its two progenitors

The chromosomes of widely cultivated hexaploid wheat (AABBDD) are derived from *Triticum turgidum* (AABB) and *Aegilops tauschii* (DD). The typical *TaTRX* genes and orthologous pairs in the two progenitors of wheat were identified based on phylogenetic relationships and sequence alignment (**Table S4**). Types of Darwin evolutionary selection are usually measured by calculating the ratio Ka/Ks (non-synonymous substitution/synonymous substitution), where Ka/Ks=1 indicates neutral selection; Ka/Ks<1 indicates purification selection; and Ka/Ks>1 indicates positive selection. KaKs Calculator software was used to calculate the Ka/Ks values of orthologous typical *TRX* genes to detect molecular selection effects. A total of 18 genes among the typical *TaTRX* genes were completely identical to the orthologous gene sequences in *Triticum turgidum* or *Aegilops Tauschii*, as shown in **Table 1**. There were 11 orthologous gene pairs with Ka/Ks values of less than 1, indicating that these genes underwent purifying selection during evolution. In addition, three pairs of orthologous genes with Ka/Ks values greater than 1 were identified, which were *TaTRX15* and *TtTRX6* (Ka/Ks=1.144), *TaTRX42* and *AeTRX11* (Ka/Ks=1.810), and *TaTRX36* and *TtTRX24* (Ka/Ks=2.615). This meant that these three typical *TaTRX* genes had undergone positive selection relative to their orthologous genes in *Triticum turgidum* or *Aegilops Tauschii*, and played important roles in the evolution process.

**Table 1:** Ka/Ks of typical *TRXs* between wheat and its two progenitors.

Ta	Tt	Ae	Result	Ta	Tt	Ae	Result
*TaTRX14*	*TtTRX5*		sequence consensus	*TaTRX43*		*AeTRX12*	sequence consensus
*TaTRX18*	*TtTRX9*		sequence consensus	*TaTRX31*		*AeTRX8*	sequence consensus
*TaTRX35*	*TtTRX17*		sequence consensus	*TaTRX23*		*AeTRX5*	Ka/Ks=0.001
*TaTRX38*	*TtTRX19*		sequence consensus	*TaTRX46*	*TtTRX22*		Ka/Ks=0.001
*TaTRX41*		*AeTRX10*	sequence consensus	*TaTRX32*	*TtTRX15*		Ka/Ks=0.001
*TaTRX13*	*TtTRX4*		sequence consensus	*TaTRX20*	*TtTRX11*		Ka/Ks=0.040
*TaTRX16*	*TtTRX7*		sequence consensus	*TaTRX40*	*TtTRX21*		Ka/Ks=0.079
*TaTRX30*		*AeTRX7*	sequence consensus	*TaTRX10*		*AeTRX2*	Ka/Ks=0.202
*TaTRX37*	*TtTRX18*		sequence consensus	*TaTRX28*	*TtTRX13*		Ka/Ks=0.323
*TaTRX17*	*TtTRX8*		sequence consensus	*TaTRX29*	*TtTRX14*		Ka/Ks=0.325
*TaTRX3*	*TtTRX1*		sequence consensus	*TaTRX34*		*AeTRX9*	Ka/Ks=0.442
*TaTRX6*	*TtTRX3*		sequence consensus	*TaTRX4*	*TtTRX2*		Ka/Ks=0.796
*TaTRX22*		*AeTRX4*	sequence consensus	*TaTRX8*		*AeTRX1*	Ka/Ks=0.846
*TaTRX47*		*AeTRX13*	sequence consensus	*TaTRX15*	*TtTRX6*		Ka/Ks=1.144
*TaTRX33*	*TtTRX16*		sequence consensus	*TaTRX42*		*AeTRX11*	Ka/Ks=1.810
*TaTRX39*	*TtTRX20*		sequence consensus	*TaTRX36*	*TtTRX24*		Ka/Ks=2.615

Ta, Triticum aestivum; Tt, Triticum turgidum; Ae, Aegilops tauschii.

### 3.6 Functional annotation analyses of typical *TaTRX* genes

Different biological databases may use different terms, making it difficult to find biological information. Gene Ontology (GO) is a system for the unified normative description of genes and gene products of all species, which has promoted the grand unification of biological information (Ashburner et al., 2000). The contents of GO include biological process (BP), molecular function (MF), and cellular component (CC). A GO analysis of typical *TaTRX* genes (**Figure S7**, **Table S5**) indicated that the BP GO term for all 48 genes was cell redox homeostasis (GO: 0045454). The BP GO term and MF GO term of 39 genes were glycerol ether metabolic process (GO:0006662) and protein disulfide oxidoreductase activity (GO:0015035), respectively. In addition, the MF GO term and CC GO term of typical *TaTRX16/18/20/23/25* also included enzyme activator activity (GO:0008047) and chloroplast stroma (GO:0009570), respectively, suggesting that these five genes may activate enzymes in the chloroplast stroma. The BP GO term for *TaTRX18* and *TaTRX23* was response to cytokinin (GO:0009735), indicating that these two genes might be involved in the cytokinine signaling pathway.

### 3.7 Relationship between the redox status and the expression of *TaTRX24*

**Figure 4:**
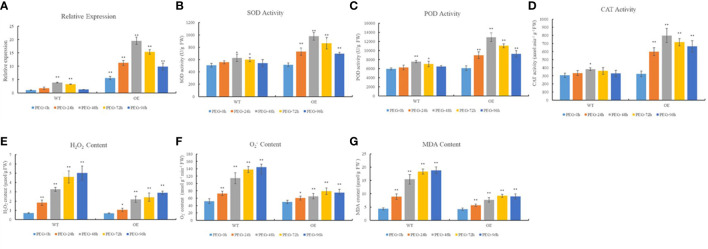
Relationship between the redox status and the expression of *TaTRX24*. **(A)** The relative expression of *TaTRX24* gene in WT and OE line before and at 24h, 48h, 72h and 96h after treatment with 20% PEG 6000. **(B)** The SOD activity in WT and OE line before and at 24h, 48h, 72h and 96h after treatment with 20% PEG 6000. **(C)** The POD activity in WT and OE line before and at 24h, 48h, 72h and 96h after treatment with 20% PEG 6000. **(D)** The CAT activity in WT and OE line before and at 24h, 48h, 72h and 96h after treatment with 20% PEG 6000. **(E)** The H_2_O_2_ content in WT and OE line before and at 24h, 48h, 72h and 96h after treatment with 20% PEG 6000. **(F)** The O_2_^-^ content in WT and OE line before and at 24h, 48h, 72h and 96h after treatment with 20% PEG 6000. **(G)** The MDA content in WT and OE line before and at 24h, 48h, 72h and 96h after treatment with 20% PEG 6000. PEG-0h, PEG-24h, PEG-48h, PEG-72h and PEG-96h respectively represent that the above wheat materials were not treated with 20% PEG 6000, and were treated with 20% PEG 6000 for 24h, 48h, 72h and 96h. Values are mean ( ± SE) of three biological replicates. *P < 0.05, **P < 0.01 represent significant difference between the WT (PEG-0h) and the others, respectively.

To confirm that *TRX* plays an important role in maintaining redox balance in wheat, we investigated the relationship between the redox status and *TaTRX24* gene expression. As shown in **Figure 4**, after PEG 6000 treatment at the seedling stage, the relative expression level of the *TaTRX24* gene in the wild type (WT) and the overexpression (OE) line showed a trend of first increasing and then decreasing, and it reached the maximum at 48 HAD. The expression level of the *TaTRX24* gene in the OE line was higher than WT at different HADs, and the OE line was 5.06 times higher than WT, especially at 48 HAD. The activities of antioxidant enzymes (SOD, POD, and CAT), and the contents of H_2_O_2_, O_2_^-^ and MDA in the leaves of the WT and OE lines also reached a peak at 48 HAD. At different HADs, the activities of antioxidant enzymes (SOD, POD, and CAT) in OE line were higher than those in WT, while the contents of H_2_O_2_, O_2_^-^, and MDA were lower than those in WT. At 48 HAD, the activities of SOD, POD, and CAT in the OE line leaves were 1.56, 1.71, and 2.09 times that of WT, respectively, while the contents of H_2_O_2_, O_2_^-^, and MDA in the OE line leaves were 67.43%, 57.15% and 49.53% of WT, respectively. These results show that after the expression level of *TaTRX24* gene was increased under dehydration stress, the activities of antioxidant enzymes (SOD, POD, and CAT) were relatively improved, while the contents of H_2_O_2_, O_2_^-^, and MDA were relatively reduced. This suggests that the *TaTRX24* plays an important role in maintaining redox balance after dehydration stress.

### 3.8 Analyses of *cis*-elements in typical *TaTRX* gene promoters

By predicting *cis*-elements in the promoters of typical *TaTRX* genes, a better understanding of the transcriptional regulation and potential functions of these genes can be obtained. The *cis*-elements of the typical *TaTRX* genes were analyzed using the 2000 bp genome sequences upstream of the typical *TRX* gene transcription initiation site in wheat and the PlantCARE online tool (**Figures S8**, **Figure S9**). The transcription related *cis*-elements, TATA-box and CAAT-box (the basic core components of the promoter), were found in all typical *TaTRX* genes. The *cis*-elements related to hormones and stresses were subsequently sorted out. The results show that there were nine kinds of elements related to hormone response, such as abscisic acid response element ABRE, gibberellin response element P-box, GARE-motif and TATC-box, auxin response element AuxRR-core and TGA-element, methyl jasmonate response element CGTCA-motif and TGACG-motif, and salicylic acid response element TCA. There were eight kinds of elements related to stress, i.e., MBS, MYB, and MYC (response to drought), LTR (response to low temperature), DRE (response to low temperature, dehydration and salt stress), ARE and GC-motif (response to hypoxia), and WUN-motif (response to wound). ABRE, TGACG-motif, CGTCA-motif, and MYC and MYB elements featured frequently in the promoters of typical *TaTRX* genes. *TaTRX4* and *TaTRX46* contained 11 and 9 ABRE elements, respectively, suggesting that these two genes might play important regulatory roles in the ABA signaling pathway. *TaTRX15* and *TaTRX24* both contained six TGACG-motif elements, and *TaTRX10* contained seven CGTCA-motif elements, suggesting that these three genes might play important roles in the methyl jasmonate signaling pathway. All the promoters of typical *TaTRX* genes contained 95 MYC elements and 75 MYB elements. In general, it could be speculated that wheat typical *TRX* genes might be widely involved in hormone metabolism and stress response, and that the *cis*-elements of different typical *TaTRX* genes might be dissimilar, suggesting that these genes might play divergent roles in wheat. Furthermore, typical *TaTRX* genes in the same subgroup might perform different functions, while genes in different subgroups might work together.

### 3.9 Expression profiles of typical *TaTRX* genes

**Figure 5:**
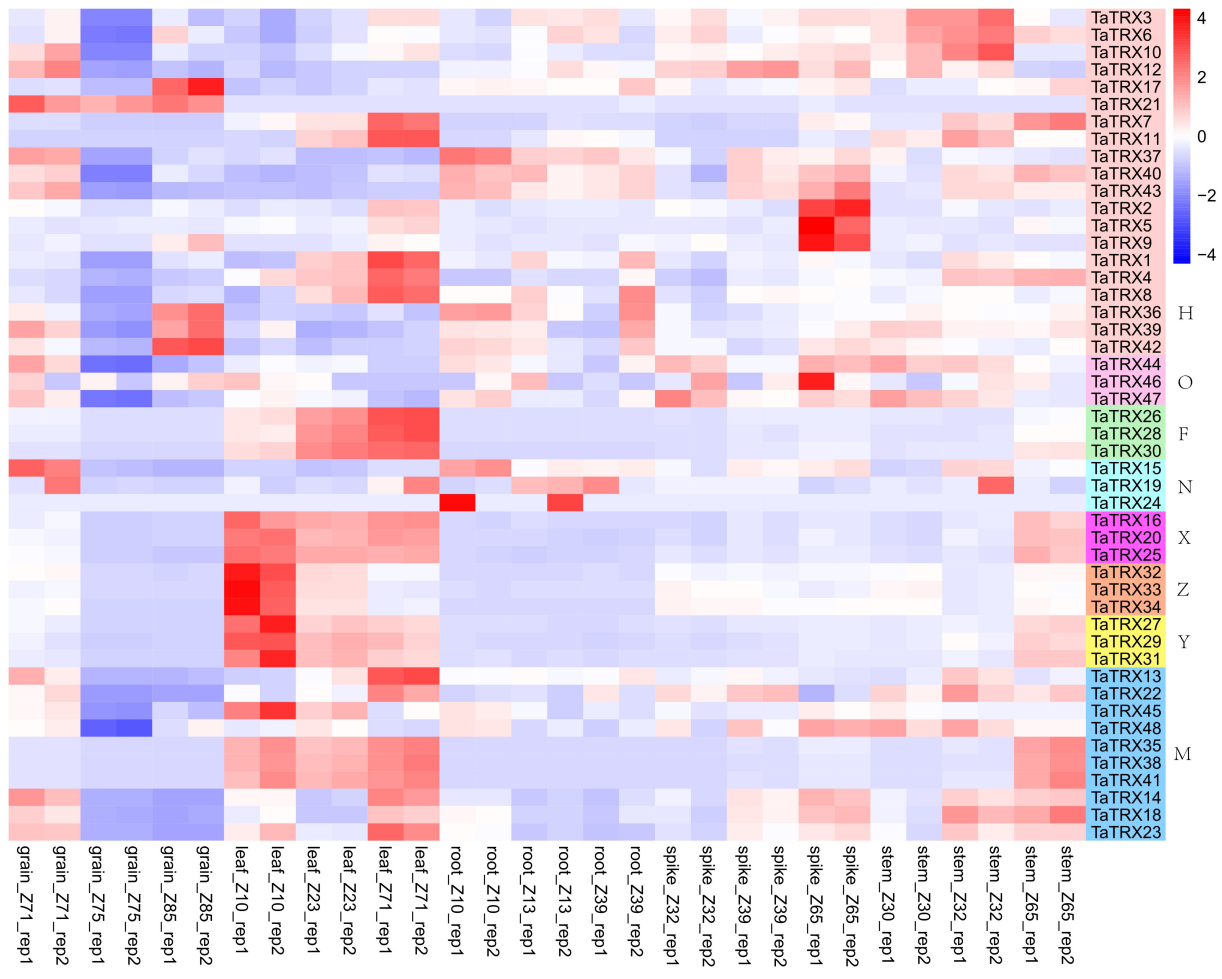
Expression profiles of typical *TaTRX* genes in different tissues and organs. grain_Z71, grain_Z75 and grain_Z85 were 2d, 15d and 30d grains after flowering, respectively. leaf_Z10, leaf_Z23 and leaf_Z71 represent leaf at seedling stage, flag leaf at tillering stage and leaf 2d after flowering, respectively. root_Z10, root_Z13 and root_Z39 represent roots at seedling stage, three-leaf stage and flag leaf stage, respectively. spike_Z32, spike_Z39 and spike_Z65 represent the spikes at two-edged stage, flag leaf stage and flowering stage respectively. stem_Z30, stem_Z32 and stem_Z65 were the ear at 1cm length, the ear at two-edged stage and the ear at flowering stage respectively. The red, white and blue cells represent the highest, medium and lowest gene expression levels, respectively. H, O, F, N, X, Z, Y, and M represent different subtypes of typical *TaTRX* genes, respectively. The colour scale represents Log2 expression values.

The analyses of gene spatiotemporal expression specificity could provide valuable information for studying the function of typical *TaTRX* genes in wheat growth and development. The RNA-seq data titled “choulet_URGI” were downloaded to analyze the spatial and temporal expression profiles of typical *TaTRX* genes in wheat from expVIP website (http://www.wheat-expression.com/). Moreover, the spatiotemporal-specific expression clustering heatmap was plotted based on log_2_TPM+1 (transcripts per million) values (**Figure 5** and **Table S6**). Compared with other genes in the family, the homologous group *TaTRX37*/*40*/*43* (H type) was highly expressed in wheat roots at the seedling stage, three leaf stage, and flag leaf stage. *TaTRX6* (H type) and *TaTRX22* (M type) were highly expressed in the stem of the ear at a 1cm length, the ear at the two-edged stage, and the ear at the flowering stage. The homologous groups of *TaTRX16*/*20*/*25* (X type), *TaTRX26*/*28*/*30* (F type), *TaTRX27*/*29*/*31* (Y type), and *TaTRX35*/*38*/*41* (M type) were highly expressed in wheat leaves at the seedling stage, the tillering stage, and 2d after the flowering stage. Compared with the two–edged stage and flag–leaf stage, the expression of *TaTRX2*/*5*/*9* (H type) increased in the spike of the flowering stage. *TaTRX21* (H type) was highly expressed in the grain of 2d, 15d, and 30d after flowering. In addition, *TaTRX14*/*18*/*23* (M type) and *TaTRX37*/*40*/*43* (H type) were highly expressed in the grain of 2d after flowering, while *TaTRX36*/*39*/*42* (H type) were highly expressed in the grain of 30d after flowering. Most of the homologous genes of typical *TaTRX* had similar spatiotemporal expression patterns. However, some homologous genes showed different expression patterns. For example, *TaTRX45* (M type) was highly expressed in the leaf, while its homologous gene *TaTRX48* was highly expressed in the stem, suggesting that these homologous genes were subfunctionalized.

**Figure 6:**
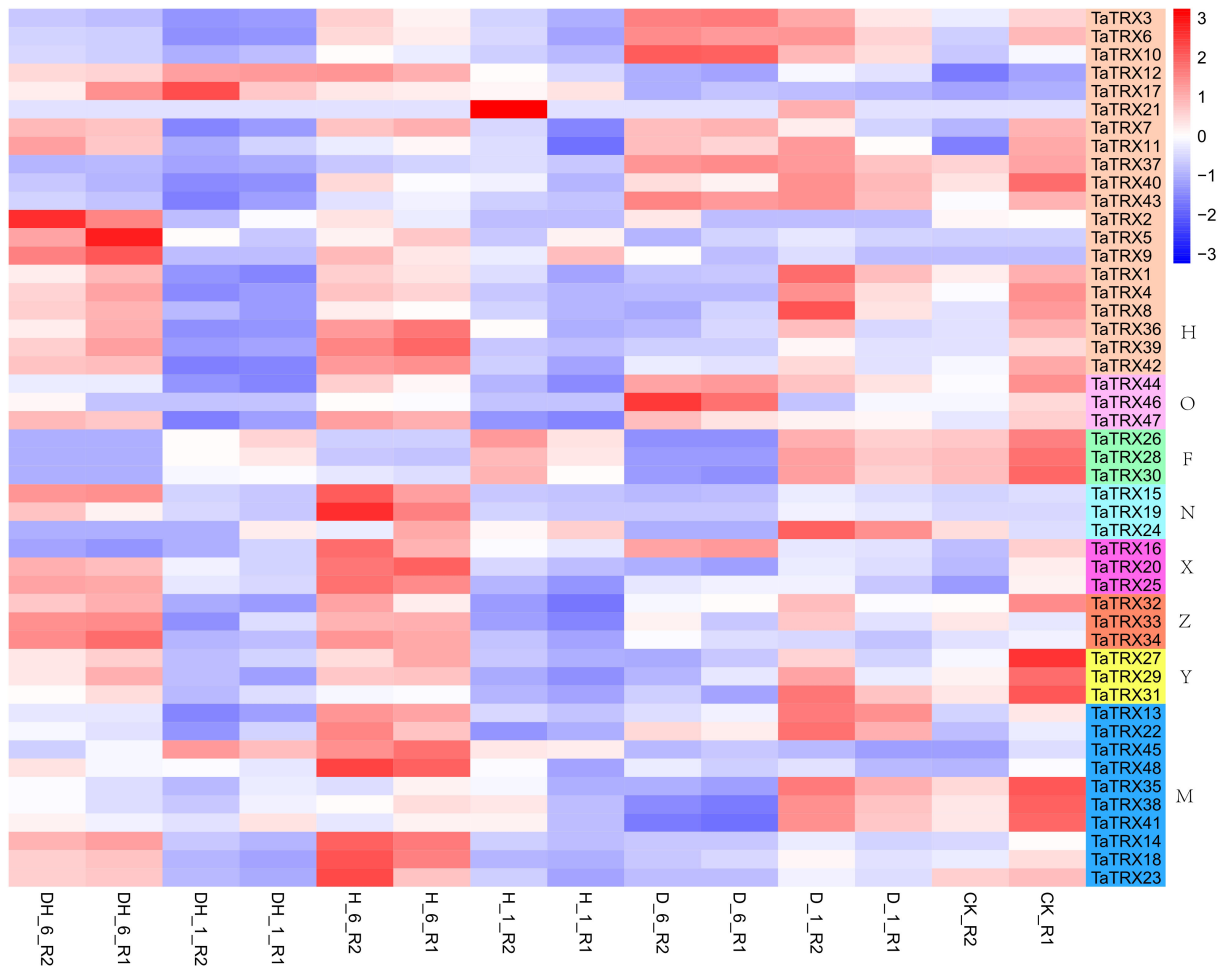
Expression profiles of typical *TaTRX* genes in wheat before and after drought stress, heat stress and co-drought and heat stress. D_1 and D_6 represent 1h and 6h after drought stress treatment of wheat respectively; H_1 and H_6 represent 1h and 6h after hot stress treatment of wheat respectively; DH_1 and DH_6 represent 1h and 6h after co-drought and heat stress treatment of wheat respectively; CK represents no stress treatment of wheat. The red, white and blue cells represent the highest, medium and lowest gene expression levels, respectively. H, O, F, N, X, Z, Y, and M represent different subtypes of typical *TaTRX* genes, respectively. The colour scale represents Log2 expression values.

**Figure 7:**
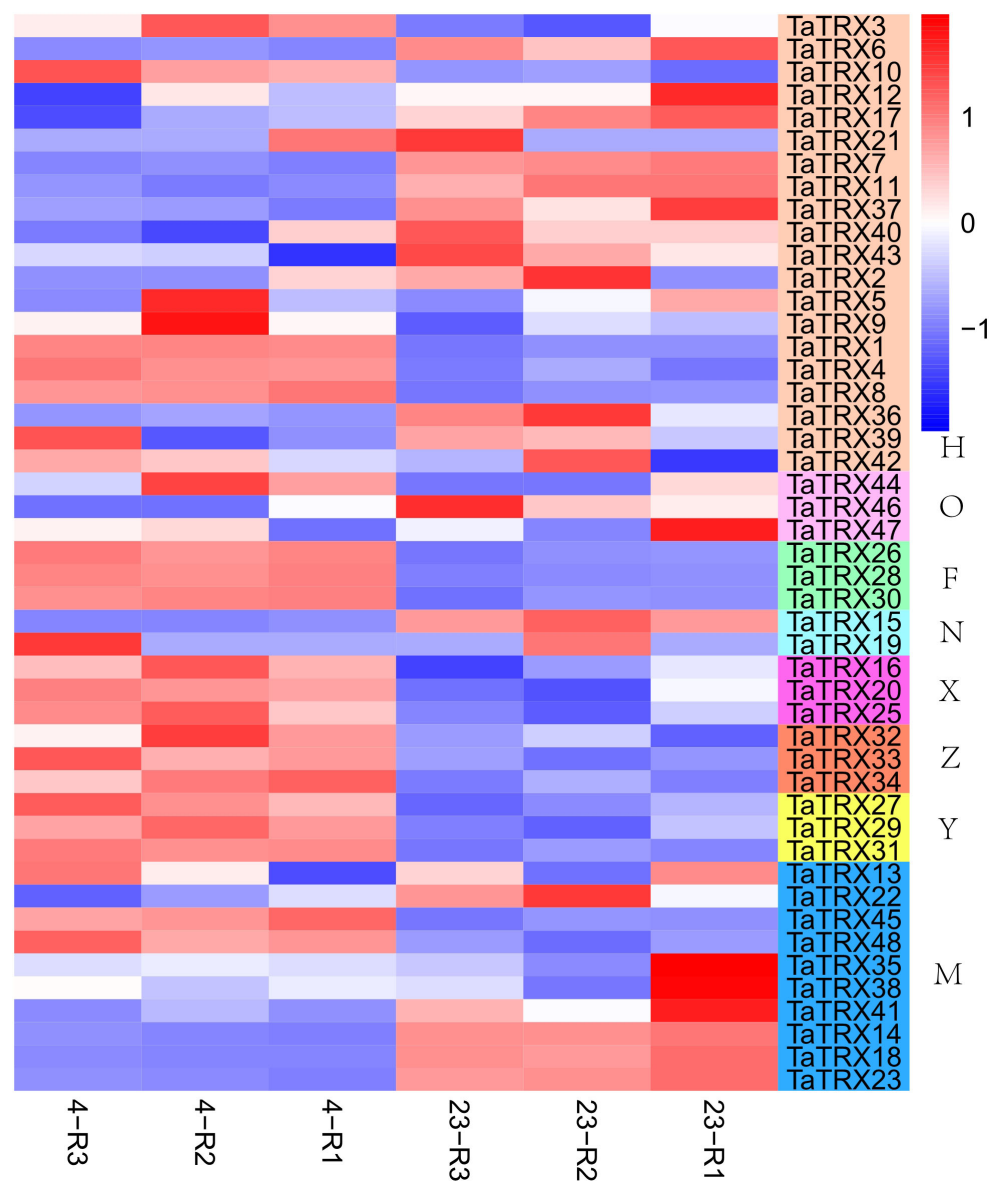
Expression profiles of typical *TaTRX* genes in wheat before and after cold stress. 23 represents wheat without cold stress treatment; 4 represents wheat treated by 4°C cold stress. The red, white and blue cells represent the highest, medium and lowest gene expression levels, respectively. H, O, F, N, X, Z, Y, and M represent different subtypes of typical *TaTRX* genes, respectively. The colour scale represents Log2 expression values.

**Figure 8:**
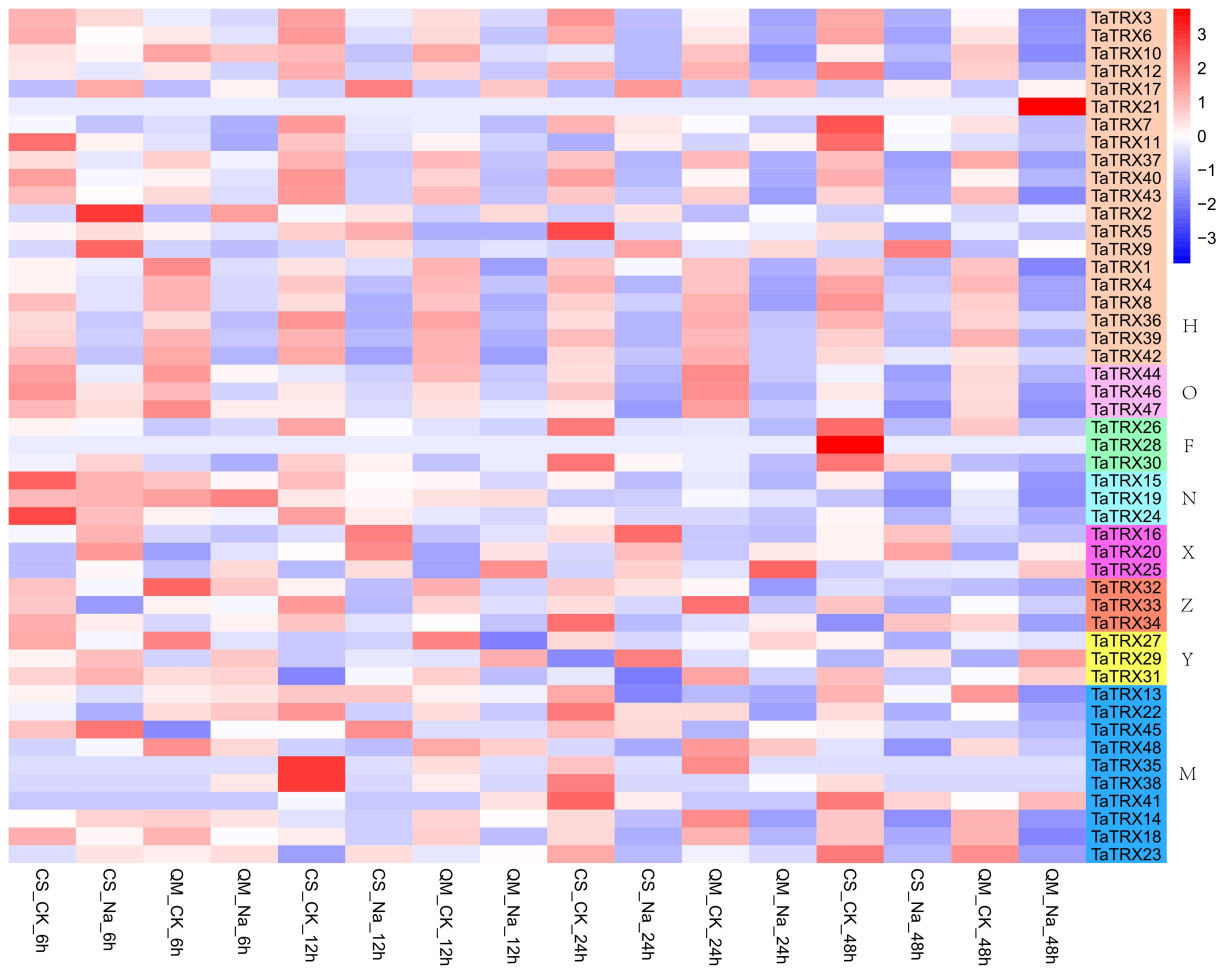
Expression profiles of typical *TaTRX* genes in wheat treated with NaCl for 6h, 12h, 24h and 48h. CS and QM represent wheat varieties “Chinese Spring” and “Qing Mai 6” respectively; CK represents no stress treatment of wheat; Na represents NaCl treatment of wheat. The red, white and blue cells represent the highest, medium and lowest gene expression levels, respectively. H, O, F, N, X, Z, Y, and M represent different subtypes of typical *TaTRX* genes, respectively. The colour scale represents Log2 expression values.

**Figure 9:**
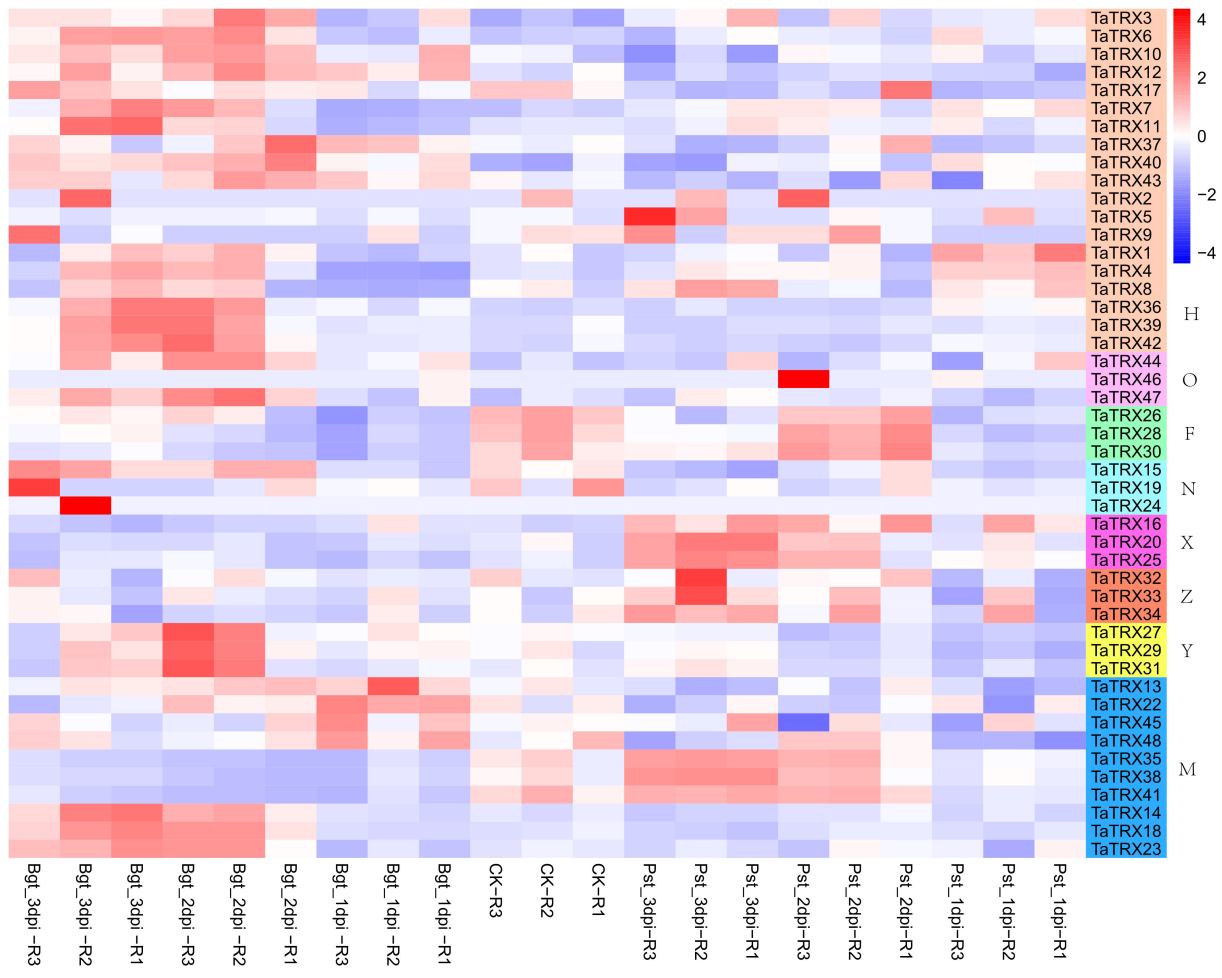
Expression profiles of typical *TaTRX* genes in wheat before and after the injection of powdery mildew and stripe rust. Bgt_1dpi, Bgt_2dpi and Bgt_3dpi represent 24h, 48h and 72h after powdery mildew pathogen was injected into wheat leaves; Pst_1dpi, Pst_2dpi and Pst_3dpi represent 24h, 48h and 72h after stripe rust pathogen was injected into wheat leaves; CK represents leaves not injected with powdery mildew and stripe rust. The red, white and blue cells represent the highest, medium and lowest gene expression levels, respectively. H, O, F, N, X, Z, Y, and M represent different subtypes of typical *TaTRX* genes, respectively. The colour scale represents Log2 expression values.

In order to understand the expression profiles of typical *TRX* genes in response to abiotic and biotic stresses in wheat, RNA–seq data (SRP045409, SRP043554, SRP062745 and SRP041017) were used to study the expression patterns of typical *TaTRXs* subjected to four abiotic stresses (drought, heat, cold, and salt) and two biotic stresses (powdery mildew and stripe rust) (**Figures 6**–**9**, **Table S6**). The expression of *TaTRX3*/*6*/*10* (H type) and *TaTRX44* (O type) increased gradually after 1h and 6h of drought stress, while the expression of two homologous groups, *TaTRX26*/*28*/*30* (F type) and *TaTRX35*/*38*/*41*(M type) decreased sharply after 6h of drought stress. The expression levels of *TaTRX12*/*17* (H type) and *TaTRX45*/*48* (M type) were gradually up-regulated after heat stress treatment for 1h and 6h, while the expression levels of *TaTRX15*/*19* (NRX) and *TaTRX16*/*20*/*25* (X type) were sharply up-regulated after heat stress treatment for 6h. The expression profiles of typical *TaTRXs* under co-drought and heat stress were similar to that under heat stress (**Figure 6**). After 4 °C cold stress treatment on wheat, the expressions of seven homologous groups, *TaTRX1/4/8* (H type), *TaTRX3/10* (H type), *TaTRX16/20/25* (X type), *TaTRX26/28/30* (F type), *TaTRX27/29/31* (Y type), *TaTRX32/33/34* (Z type), and *TaTRX45/48* (M type), were significantly up-regulated. However, *TaTRX6* (H type), which was the homologous gene of *TaTRX3/10*, was down–regulated (**Figure 7**). After salt (NaCl) stress treatment, the expressions of *TaTRX16/20/25* (X type) were gradually up-regulated at 6h, 12h, and 24h, and down–regulated at 48h. The expressions of *TaTRX2/9* (H type) were gradually down–regulated after 6h, while their homologous gene *TaTRX5* was down–regulated after 12h. The expression of *TaTRX17* (H type) was up-regulated in all stages after salt stress treatment, while its homologous gene *TaTRX12* was down–regulated in all stages (**Figure 8**).

As shown in **Figure 9**, after infecting wheat leaves with stripe rust, compared with healthy leaves, the expressions of *TaTRX1/4/8* (H type) were up-regulated at 24h, and then the expressions of *TaTRX1/4* were continuously down–regulated, while the expression of *TaTRX8* was down–regulated at 48h, and then up–regulated at 72h. The gene expressions of *TaTRX16/20/25* (X type) were continuously up–regulated at 48h and 72h, while the expressions of *TaTRX35/38/41* (M type) were down–regulated at 24h and then up–regulated at 48h and 72h. After infecting wheat leaves with powdery mildew, the gene expressions of *TaTRX13/22* (M type) were up–regulated at 24h, and then decreased compared with healthy leaves. *TaTRX37/40/43* (H type) expressions were up–regulated at 24h and 48h, and then down–regulated at 72h; the expressions of *TaTRX1/4/8* (H type), *TaTRX3/6/10* (H type), *TaTRX7/11* (H type), *TaTRX14/18/23* (M type), *TaTRX27/29/31* (Y type), and *TaTRX36/39/42* (H type) increased at 48h and 72h. The expressions of *TaTRX35/38/41* (M type) were down–regulated at 24h, 48h, and 72h. In conclusion, only *TaTRX1/4/8* homologous group could be up–regulated when wheat leaves were infected with stripe rust and powdery mildew respectively. A total of eight typical *TaTRX* homologous groups were up–regulated in response to infection with powdery mildew, and most of them were H type, while only three homologous groups were up–regulated after the infection of stripe rust.

### 3.10 Validation of the expressions of typical *TaTRX* genes using RT-qPCR analyses

**Figure 10:**
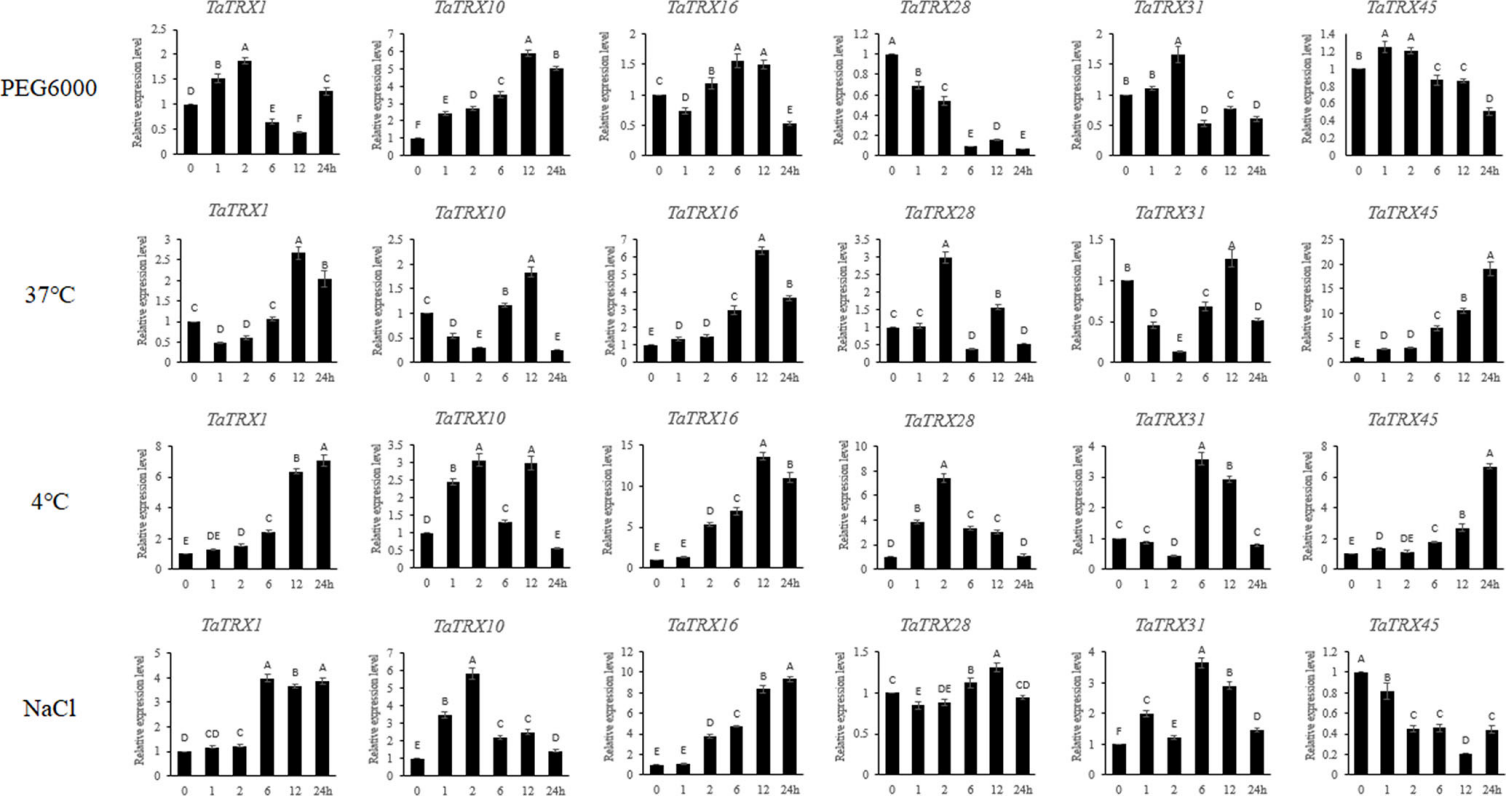
Expression analyses of typical *TaTRX* genes in wheat variety Chinese Spring (CS) under different treatments by RT-qPCR. Relative expression levels of typical *TaTRXs* in response to PEG 6000 (20%), heat (37°C), cold (4°C) and NaCl (200 mM) for 0 h, 1 h, 2 h, 6 h, 12 h, and 24 h in the leaves at the two-leaf stage. Data were normalized with *β-actin* gene. Vertical bars indicate standard deviations. Different capital letters indicate extremely significant differences at *p* < 0.01 according to one-way ANOVA and *post-hoc* Tukey’s test.

In order to verify the reliability of the above transcriptome data, six typical *TaTRX* genes were randomly selected and their expressions at multiple time points under different stress conditions were analyzed *via* RT-qPCR. The overall expression trends of these genes obtained *via* RT-qPCR analysis were basically consistent with that of RNA-seq analyses (**Figure 10**). In detail, under dehydration stress simulated using 20% PEG 6000, the expression levels of *TaTRX1*, *TaTRX31*, and *TaTRX45* all showed a trend of increasing at first and then decreasing, and they reached the highest expression levels at 2h, 2h, and 1h, respectively. The expression of *TaTRX10* showed a gradual upward trend and reached the highest level at 12h. Conversely, the expression of *TaTRX28* showed a gradual downward trend with the increase in treatment time. Under 37°C heat stress treatment, the expressions of *TaTRX1*, *TaTRX10* and *TaTRX31* showed a trend of decreasing first and then increasing, while the expression of *TaTRX16* and *TaTRX45* showed a gradual increasing trend with the extension of treatment time. Under the treatment of 4°C cold stress, the expression of *TaTRX1*, *TaTRX16* and *TaTRX45* showed a gradually increasing trend, while the expression of *TaTRX28* reached the highest level at 2h and gradually decreased, and the expression of *TaTRX31* reached the highest level at 6h. Under NaCl stress, the expression of *TaTRX16* gradually increased and reached a maximum at 24h, while the expression of *TaTRX45* gradually decreased, and the expression of both *TaTRX1* and *TaTRX31* reached a maximum at 6h. These results meant that the transcriptome data used above were reliable.

### 3.11 Protein interaction networks analyses of typical TaTRXs

The orthology-based method was used to predict the interaction proteins of typical TaTRXs since the protein interaction database of wheat is not perfect, so as to better understand the biological function and mechanism of this family in wheat. A total of 16 proteins interacting with typical TaTRXs were predicted and named according to their homologies (**Figure S10**, **Table S8**). Among these 16 interacting proteins, TaNTR1-A, TaNTR1-B, and TaNTR2-D belonged to the NADPH-dependent thioredoxin reductase (NTR) family in wheat, TaFTR1-A and TaFTR1-D belonged to the ferredoxin-thioredoxin reductase (FTR) family in wheat, TaPrx1-A, TaPrx1-B, TaPrx1-D, TaPrx2-B, and TaPrx2-D belonged to the peroxiredoxin (Prx) family, and TaFRK1-A, TaFRK2-A, TaFRK2-B, TaFRK2-D, TaFRK3-A, and TaFRK3-B were members of the fructokinase (FRK) family. NTR, FTR, and Prx play important roles in the wheat redox regulation pathway. Only three Z-type TRXs (TaTRX32, TaTRX33, and TaTRX34) can interact with the above six TaFRK proteins, suggesting that Z-type TaTRXs might be able to participate in regulating the glycolysis pathway of wheat by regulating the redox states of TaFRKs, while other subtypes of TaTRXs should have no such function.

## 4 Discussion

TRXs are important proteins that are widely present in organisms, and they regulate the redox state of cells. The TRX system in plants is widely involved in transcription and translation, metabolism, signal transduction, and so on, under normal circumstances (Geigenberger et al., 2017). When plants suffer from biotic or abiotic stresses, the concentration of ROS increases, and plants will be in a state of oxidative stress, resulting in serious damage to biological macromolecules such as DNA, proteins, and lipids (Miller et al., 2010). The TRX system can reduce, repair, and refold proteins that are damaged by oxidation in the body to restore their activity (Meyer et al., 2009; Wang et al., 2010), regulate the balance of other redox systems in the body, such as the glutathione (GSH) system, and maintain the stability of the redox balance of various physiological and biochemical processes in plants (Kanzok et al., 2001). The genome-wide analyses of the *TRX* family has been widely carried out because the genomes of many species have been sequenced in recent years. In total, 41, 61, 48, and 150 *TRX* family members have been identified in Arabidopsis (Chibani et al., 2021), rice (Nuruzzaman et al., 2012), grape (Zhang J. R. et al., 2021), and upland cotton (Elasad et al., 2018), respectively. However, due to the complexity of the wheat genome, the current research on wheat *TRX* family genes is incomplete. In this study, we conducted genome-wide identification and multifaceted analyses of typical *TRX* family members in wheat. This study provides a foundation for better understanding the roles of typical *TaTRX* members in wheat growth and stress responses.

In this study, a total of 48 typical *TaTRX* gene family members were identified by searching at the whole genome level of wheat, of which 16, 15, and 17 typical *TaTRX* genes were distributed in the A, B, and D subgenomes, respectively, indicating that there was no significant change in the gene abundance of typical *TaTRX* genes at the subgenome scale. Among the 17 homoeological groups, three groups contained only two homoeological copies, indicating that homologous copy loss events might occur in the typical *TRX* gene family of wheat during wheat polyploidy. The number of typical *TRX* members in Arabidopsis, rice, and maize were about 41.7%, 35.4% and 41.7% of that in wheat, respectively, indicating that allohexaploization was conducive to the expansion of typical *TRX* family members in wheat. The key to the increase in the diversity of gene family members usually includes gene tandem duplication and segmental duplication events. The members of the gene family formed by tandem replication are usually closely arranged on the same chromosome, forming a gene cluster with similar sequence and function. Fragment duplication events are large-scale gene proliferation processes at the chromosome level (Schmutz et al., 2010; Su et al., 2020). A study on the amplification of soybean *ANK* gene family members showed that the contribution rates of tandem duplication and segmental duplication events were 35.8% and 46.0%, respectively (Zhao et al., 2020); about 10.5% and 64.8% of wheat *SCPL* family genes were identified from tandem duplication and segmental duplication events, respectively (Xu et al., 2021). In this study, consistent with these prior findings, 8.3% of the members of the typical *TRX* family in wheat were shown to originate from tandem duplication, and 83.3% from segmental duplication, indicating that segmental duplication was the main factor in the amplification of the typical *TRX* genes in the wheat genome. The expansions of gene families are usually considered to be the result of species evolution, which helps species to improve their adaptabilities in different environments, so that they are widely distributed (Theiben et al., 2016; Zhao et al., 2021). The analyses of collinearity among species can provide information for the study of genome evolution history (Wang et al., 2012). In this study we found that 15 typical *TRX* genes in wheat, seven typical *TRX* genes in rice, and 11 typical *TRX* genes in maize might have common genetic origins, suggesting that these orthologous pairs might have existed before the differentiation of monocotyledon ancestors.

The codon system encoding amino acids has the property of degeneracy, and in general, a change in the third base causes a synonymous substitution, while a change in the first or second base causes a non-synonymous substitution. In genetics, Ka and Ks represent the non-synonymous replacement rate and synonymous replacement rate of two genes, respectively. Because non-synonymous substitution will cause amino acid changes, which may change the conformation and function of proteins, it will eventually lead to adaptive changes. Synonymous substitution does not change the composition of a protein, and so it is not affected by natural selection. The ratio of Ka and Ks can determine whether there is selective pressure on the coding gene of this protein (Akhunov et al., 2013). Lei et al. (2021) used PAML software to calculate the Ka/Ks values of wheat *PYL* genes and their orthologous genes of two ancestral species, and no positive selection genes were found, indicating that wheat *PYL* genes experienced purification selection in the process of evolution, and are highly conservative. Ru et al. (2021) also reached a similar conclusion in their study on wheat *DEAD-box* family genes. Ding et al. (2018) found in their study of *TCP* family genes in wheat that *TaTCP16-A* was the result of positive selection relative to its direct homologous genes in Urartu wheat (*Triticum urartu* L.), while other genes were the result of purified selection. In this study, three typical *TRX* genes of wheat were found to be positively selected relative to their orthologous ancestral species, suggesting that these three genes might play significant roles in the evolution of wheat. A total of 37.5% of the genes in the *TRX* family were identical to the orthologous gene sequences of their ancestors, indicating that the typical *TRX* family genes in wheat were conserved in the evolutionary process.

Gene structure and protein conserved motifs can provide important information for analyzing the evolutionary relationship and phylogenetic characteristics of gene families (Zhu et al., 2020), and their diversities are closely related to the evolutionary characteristics and functions of gene family members. In this study, among the typical *TRX* family genes in wheat, the gene structures of the same branch were relatively similar, but there were also a few exceptions (**Figure 2**). Structural differences can produce homologous genes with different functions, which is common in gene families (Xu et al., 2012; Zhao et al., 2021). Most proteins of the same types in the typical TRX family of wheat contained similar conserved motifs, but there were a few exceptions. For example, although TaTRX13 and TaTRX22 were in the same branch, motif3 only existed in TaTRX22, which might be related to the different compositions and arrangements of introns and exons. In addition, the putative 3D structures of the typical TRX proteins of the same subtypes were similar. In all the predicted models, the active “WCGPC” site associated with target protein reduction was located between the second *α*-helix and the second *β*-fold, these results provide important information for better understanding the biological functions of typical *TRX* genes in wheat.

The spatiotemporal expression specificity of genes will provide useful information for understanding their functions in growth and development (Tong et al., 2013). In this study, there were four typical *TaTRX* homologous groups (12 genes) with high levels of expression in wheat leaves at the three stages of seedling, tillering, and 2d after flowering stage. These 12 genes belonged to chloroplast *TRXs* and were predicted to be located in the chloroplasts. In addition, *TaTRX32/33/34* (Z type) were highly expressed in the leaves at the seedling stage and the flag leaves at the tillering stage of wheat. The research of Arsova et al. (2010) shows that the interaction between the Z type TRX and two fructokinase-like proteins (FLNs) in chloroplasts may participate in the regulation of plastid-encoded polymerase (PEP)–dependent chloroplast transcription, and then link the transcriptional regulation and light signals through photosynthetic electron transport (PET), which may have an important impact on the normal development of chloroplasts. The prediction results of the protein interaction network in this study showed that only Z type TaTRX proteins could interact with fructokinase (FRK) proteins in wheat. Bhurta et al. (2022) predicted the possible interaction proteins of 15 previously screened TaTRX proteins, and the results did not include TaFRK, which may be because these 15 TaTRX proteins do not contain Z type TRX. The target proteins of plant typical TRXs in chloroplasts may also include chlorophyll a/b binding protein, photosystem I reaction center subunit N, light harvesting complex II protein kinase, etc. (Balmer et al., 2006; Schürmann and Buchanan, 2008; Montrichard et al., 2009). Therefore, the above 15 typical *TaTRX* genes may play important roles in the absorption of light energy and the energy transfer of plants. *TaTRX14/18/23* (M type) and *TaTRX37/40/43* (H type) were highly expressed in the early stage of wheat grain development; the expressions of *TaTRX36/39/42* (H type) were higher in the late stage of wheat grain development; and the expression of *TaTRX21* (H type) was higher at different stages of grain development after anthesis, but lower in other tissues of wheat. Wong et al. (2004) used both monobromobimane (mBBr) fluorescent gel electrophoresis and mutant thioredoxin affinity column chromatography methods to identify the target proteins of TRXs during wheat grain development. These target proteins mainly include aconitase, which is involved in the tricarboxylic acid cycle; ADP glucose pyrophosphorylase (AGPase), which plays an important role in starch synthesis; enolase and triosephosphate isomerase (TPI), the key enzymes in the glycolysis pathway; and protein disulfide isomerase (PDI), etc. These proteins are involved in biochemical processes such as carbohydrate metabolism, nitrogen metabolism and protein synthesis and degradation during wheat grain development. Therefore, in this study, the above 10 typical *TaTRX* genes (most of them are H type), might play important roles in the material synthesis and metabolism of wheat grain development. Kobrehel et al. (1992) found that thioredoxin H functions as a signal of the mobilization of storage proteins to enhance the metabolic processes associated with the germination of wheat seeds. Barley seeds with Trx H overexpressed in the endosperm showed accelerated germination and early or enhanced expression of associated enzymes (α-amylase and pullulanase). The underexpression of *Trxh9* in wheat led to effects that were opposite to those observed for the overexpression of *Trxh5* in the barley-retardation of germination, and the delayed or reduced expression of associated enzymes. Wheat lines with underexpressed *Trx* showed delayed preharvest sprouting when grown in the greenhouse or in a field without a decrease in final yield (Li et al., 2009). The expression of protein disulfide isomerase in the thioredoxin s antisense transgenic wheat was up-regulated, which induced easily forming glutenin macropolymers and the resistance of storage proteins to degradation (Guo et al., 2013). The results are further evidence that the level of Trx H in cereal endosperm determines the fundamental properties as well as the potential applications of the seed.

There are usually many *cis*-acting elements that are involved in various pathways in the promoter regions of genes, and their complex interactions with trans-acting factors can regulate gene expression (Hu et al., 2016). In this study, analyses of the expression profiles of typical *TaTRXs* showed that most genes could be induced by different stress treatments, but so far, there are few studies on the *cis*-acting elements of typical *TaTRX* promoters. In this study, the promoter regions of *TaTRX3/6/10* (H type) and *TaTRX44* (O type), which were up-regulated by drought treatment, all contained the drought-responsive elements MBS, MYB, or MYC; the seven homoeologous groups that were up-regulated by cold stress treatment all contained one or more low temperature response elements, LTR or DRE; the promoter regions of *TaTRX2/5/9* (H type), *TaTRX20/25* (X type), and *TaTRX17* (H type), which were up-regulated after salt stress treatment, all contained the salt stress response-related elements DRE or MYB; these indicated that the expressions of typical *TaTRXs* after stress treatments might be affected by the interaction between their promoter-associated *cis*-acting elements and transcription factors. The promoter regions of most typical *TaTRX* genes contained ABRE elements, especially *TaTRX4* (H type) and *TaTRX46* (O type), which contained 11 and 9 ABRE elements, respectively. It is speculated that members of this family might be widely involved in wheat ABA signal transduction. Cheng et al. (Cheng et al., 2021) studied the promoter of *TaNRX1* (*TaTRX19* in this study) and showed that a 36 bp fragment containing two ABRE *cis*-acting elements was the key sequence of the *TaNRX1* gene in response to polyethylene glycol (PEG) and ABA.

The results of the GO analysis show that the biological processes of all typical *TaTRX* genes comprised cell redox homeostasis. Our research on the expression of the *TaTRX24* gene and the redox status of wheat under drought stress provides strong evidence for the results of GO. Previous studies have also shown that TRX can restore the activity of the target protein by reducing the disulfide bond that was incorrectly formed, so as to maintain the redox balance of cells (Gelhaye et al., 2005; Daloso et al., 2015). In this study, using RNA-seq data combined with RT-qPCR experiments, the expression characteristics of typical *TaTRX* genes under various biotic and abiotic stresses were deeply analyzed. The results show that different typical *TaTRX* genes can respond to different stresses and then up-regulate their expressions. For example, typical *TaTRX1/4/8* (H type) can be up-regulated after infection with stripe rust, powdery mildew, and cold stress; typical *TaTRX3/6/10* (H type) can be up-regulated after drought stress and after infection with powdery mildew; the typical *TaTRX16/20/25* (X type) can be up-regulated after exposure to cold and salt stresses, and infection with stripe rust. The target proteins of TRXs that have been identified by predecessors that can help plants resist adversity stress include superoxide dismutase (SOD), peroxidase (POD), catalase (CAT), heat shock protein (HSP), cold shock protein (CSP), betaine aldehyde dehydrogenase (BADH), etc. (Balmer et al., 2004; Alkhalfioui et al., 2007; Hägglund et al., 2008). POD is an enzyme that uses H_2_O_2_ as an electron acceptor to catalyze the oxidation of the substrate. POD is important for the removal of excess ROS in plants. In this study, several TaPrx proteins, which are thiol peroxidases, were predicted to interact with TaTRXs based on the protein interaction network database. The prediction made by Bhurta et al. (2022) on the interaction proteins of 15 TaTRXs also reached the same conclusion. The functions of these target proteins are mainly related to the scavenging of excess ROS in plant cells, the maintenance of cell osmotic potential, and the stabilities of nucleic acids, proteins, and the structures of cell membranes under stresses. Zhang et al. (2015) found that under stress conditions, AtTRX-M2 can reduce ROS generation by targeting AtVDAC3. Kneeshaw et al. (2017) showed that enzymes of the hydrogen peroxide (H_2_O_2_) scavenging pathway, including CATs, were able to suffer oxidative damage in ROS-rich environments, then NRX was able to target CAT to optimize its enzymatic activity, thereby enhancing the ability of plants to scavenge ROS. When TRX reduces the target protein, it will become oxidized, and then thioredoxin reductase (TrxR) can transfer electrons to the oxidized TRX to restore its ability to reduce the target protein (Arnér and Holmgren, 2001). Because of the important role of TrxR, Bhurta et al. (2022) and the authors of this paper all predicted that TaTRX could interact with TaTrxR by using the protein interaction network database.

In addition, genes such as *TaTRX5*, *TaTRX6*, and *TaTRX12* were differentially expressed after stress treatment compared with their homologous genes, suggesting that the typical wheat *TRX* gene family underwent the subfunctionalization of homologous genes during the process of allopolyploidization. However, the gene structures and protein conserved motifs of homologous genes in this family were not significantly different, so different UTR lengths and different distributions of *cis*-acting elements in the promoter regions might regulate the expression specificities of these homologous typical *TaTRX* genes. Further research on the potential effects of subfunctionalization for the typical *TRX* family genes in wheat will help to better mine these genes and to understand their functions and roles in wheat.

## Data availability statement

The original contributions presented in the study are included in the article/**Supplementary Material**. Further inquiries can be directed to the corresponding authors.

## Author contributions

JZ, JX and XZ conceived and designed this research; JZ performed the experiment; JZ, TS, HZ, MZ and NL analyzed the data; JZ wrote the manuscript; JZ, TS, HZ, MZ, NL, JX and XZ completed the writing review and editing. All authors contributed to the article and approved the submitted version.
