# Supplementary material for: Genome-wide identification, characterization, evolution, and expression pattern analyses of the typical thioredoxin gene family in wheat (Triticum aestivum L.)
Source: Front Plant Sci. 2022 Dec 22;13:1020584. doi: 10.3389/fpls.2022.1020584 (PMC9813791; doi:10.3389/fpls.2022.1020584)
Supplement: Supplementary file 1 [file DataSheet_1.docx]

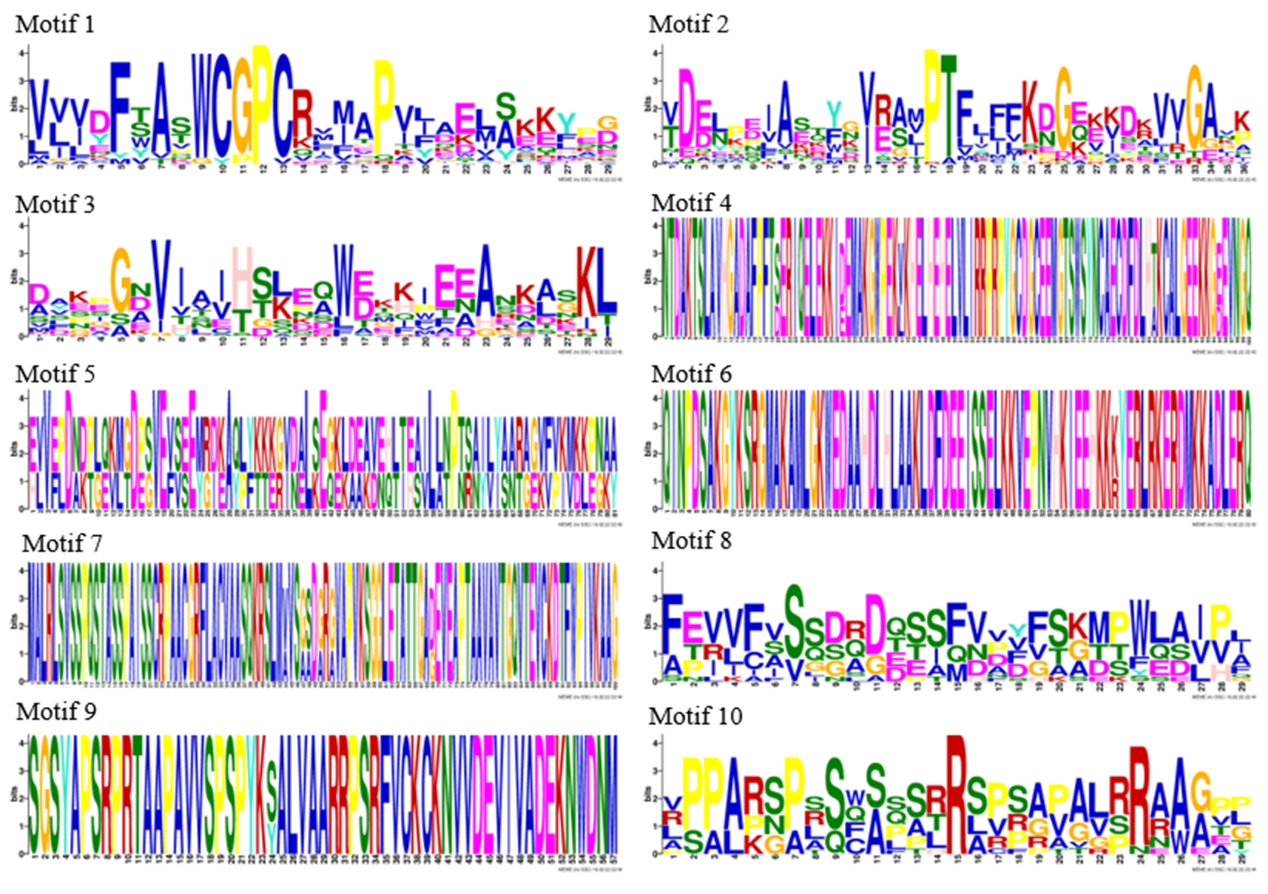


**Figure S1**. Conserved amino acid motif logos identified in these 48 typical *TaTRX* genes.


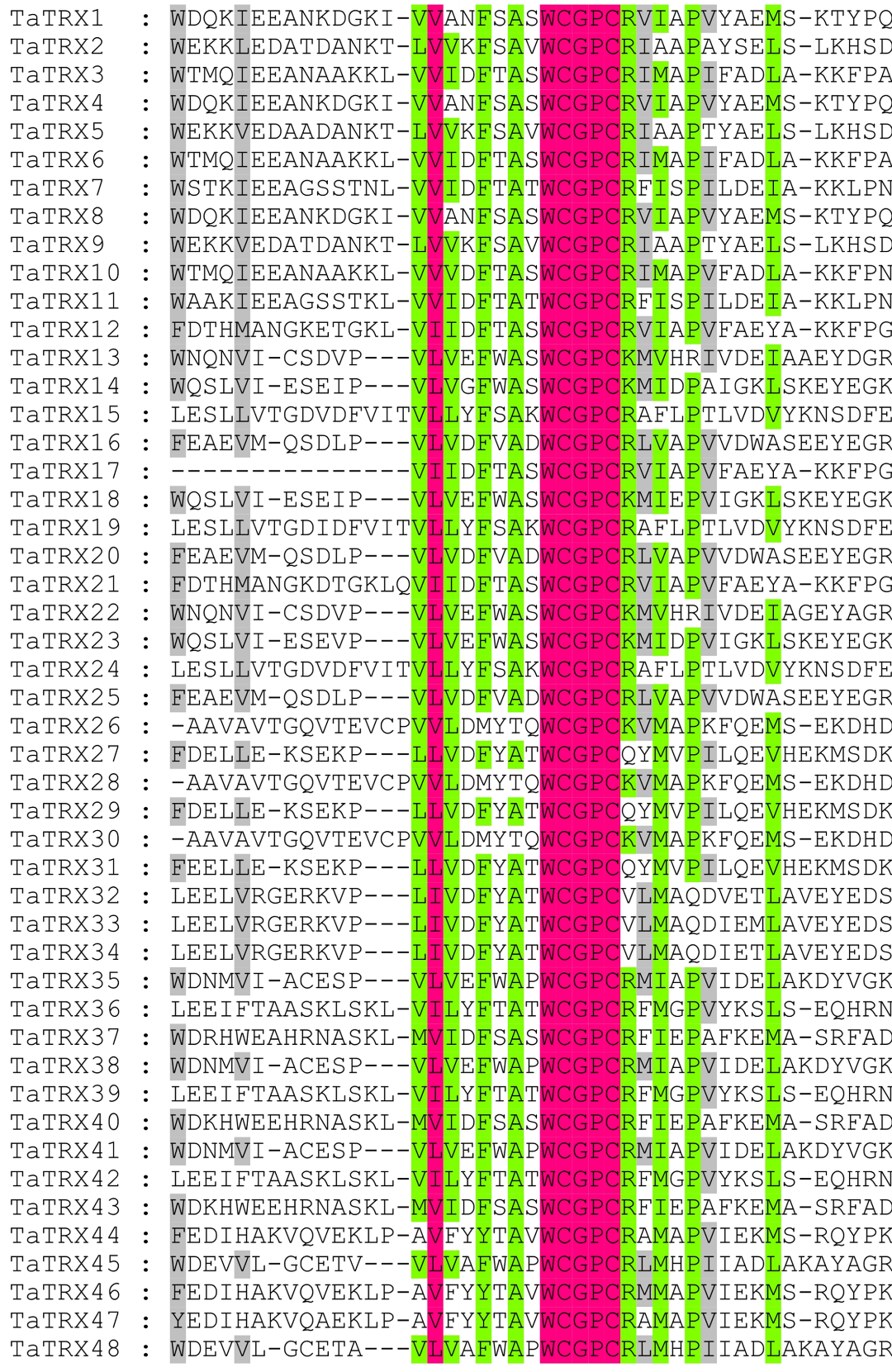


Thioredoxin domain

**Figure S2**. Multiple alignment of the thioredoxin domains from the typical TaTRX proteins based on the genedoc software.


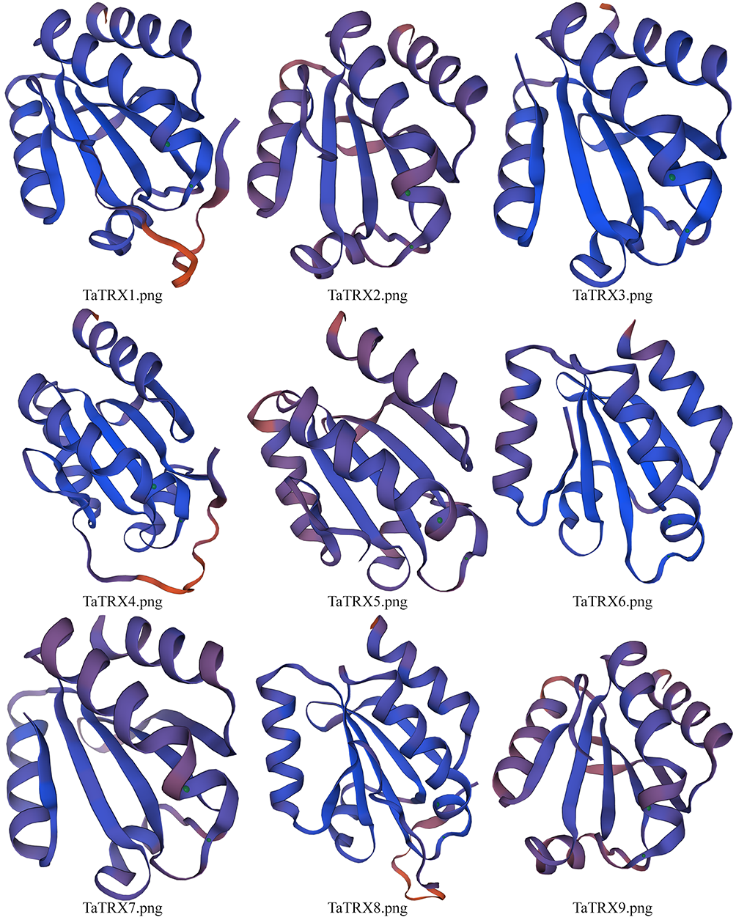


**Figure S3a**. The 3D structure modeling of typical TaTRX proteins (TaTRX1-TaTRX9). The structure images were generated using the pymol software.


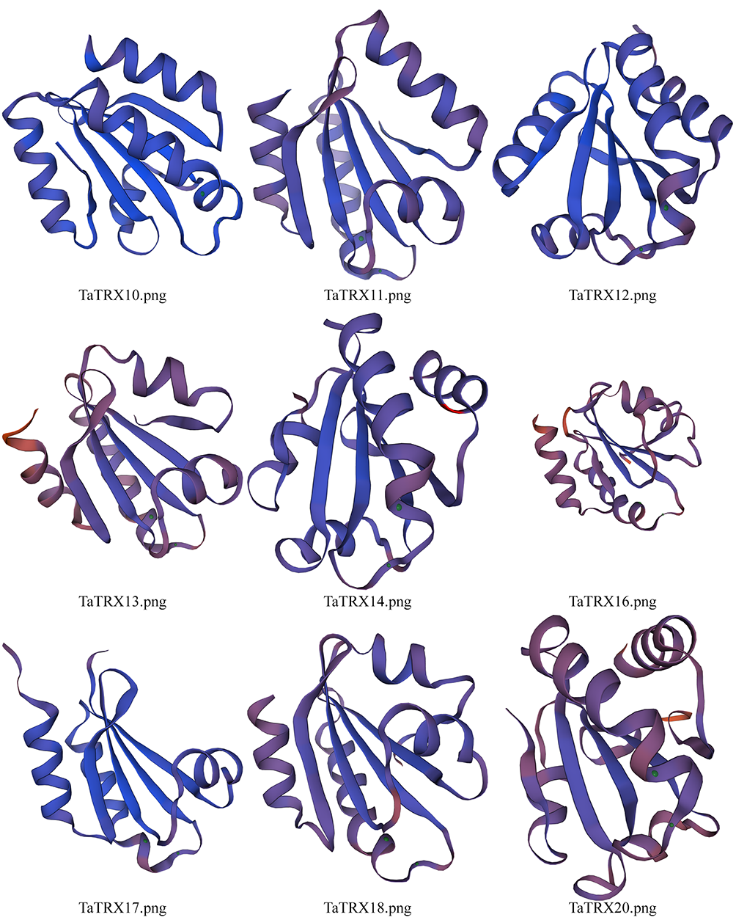


**Figure S3b**. The 3D structure modeling of typical TaTRX proteins (TaTRX10-TaTRX20). The structure images were generated using the pymol software.


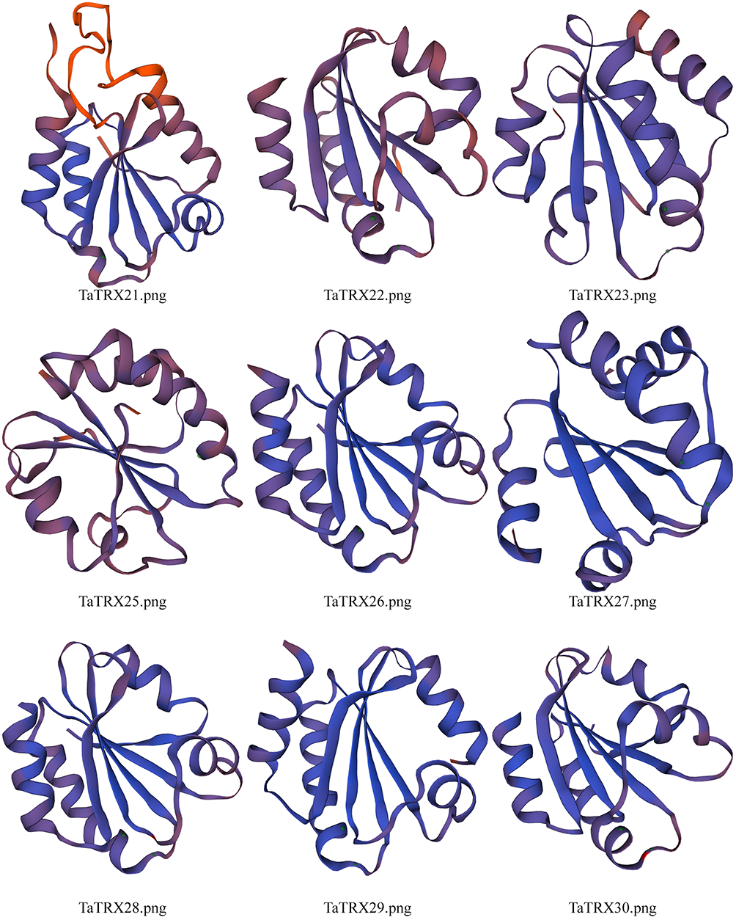


**Figure S3c**. The 3D structure modeling of typical TaTRX proteins (TaTRX21- TaTRX30). The structure images were generated using the pymol software.


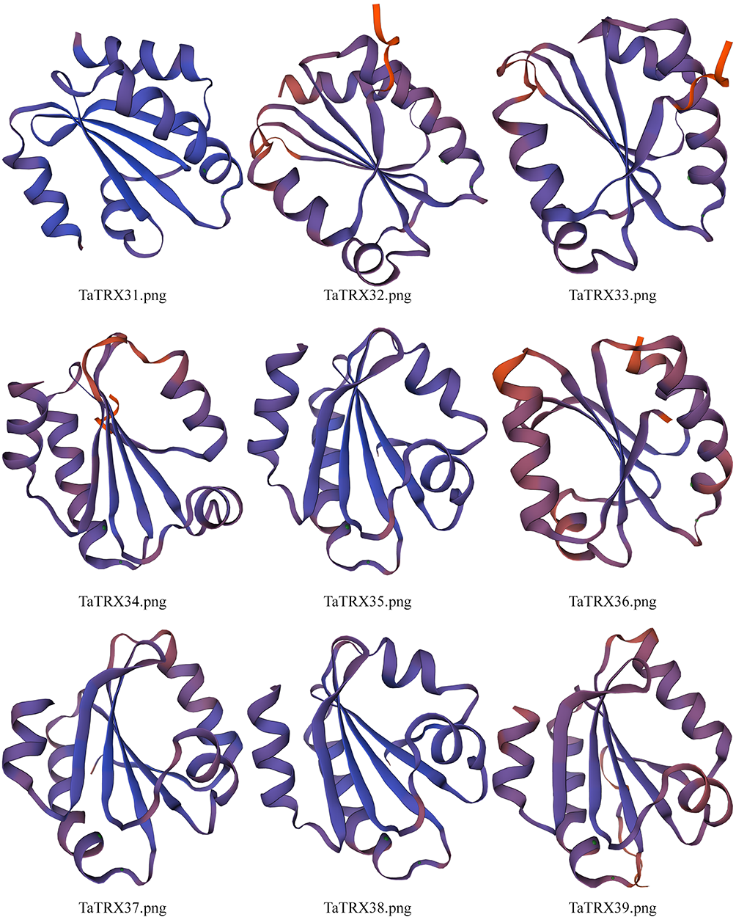


**Figure S3d**. The 3D structure modeling of typical TaTRX proteins (TaTRX31- TaTRX39). The structure images were generated using the pymol software.


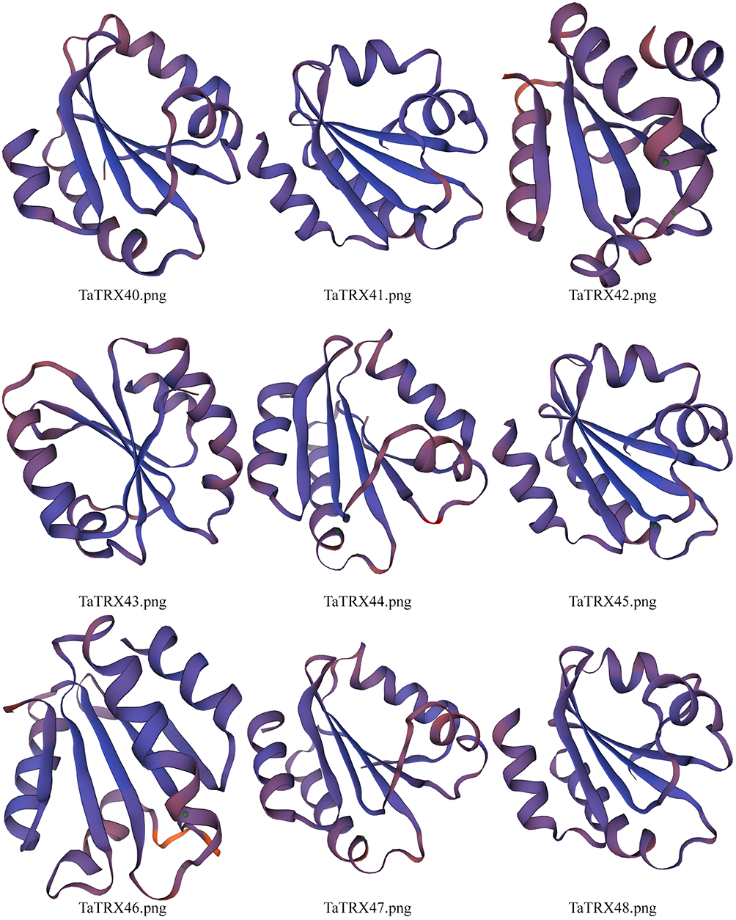


**Figure S3e**. The 3D structure modeling of typical TaTRX proteins (TaTRX40- TaTRX48). The structure images were generated using the pymol software.


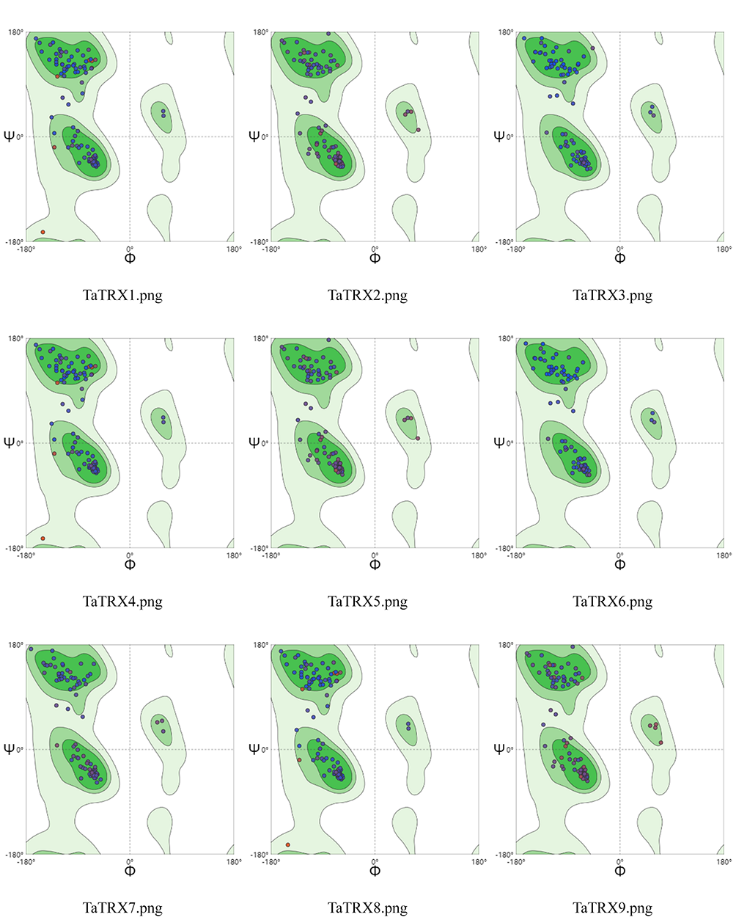


**Figure S4a**. Calculated ramachandran plots for modeled 3D structures of typical TaTRX proteins (TaTRX1-TaTRX9).


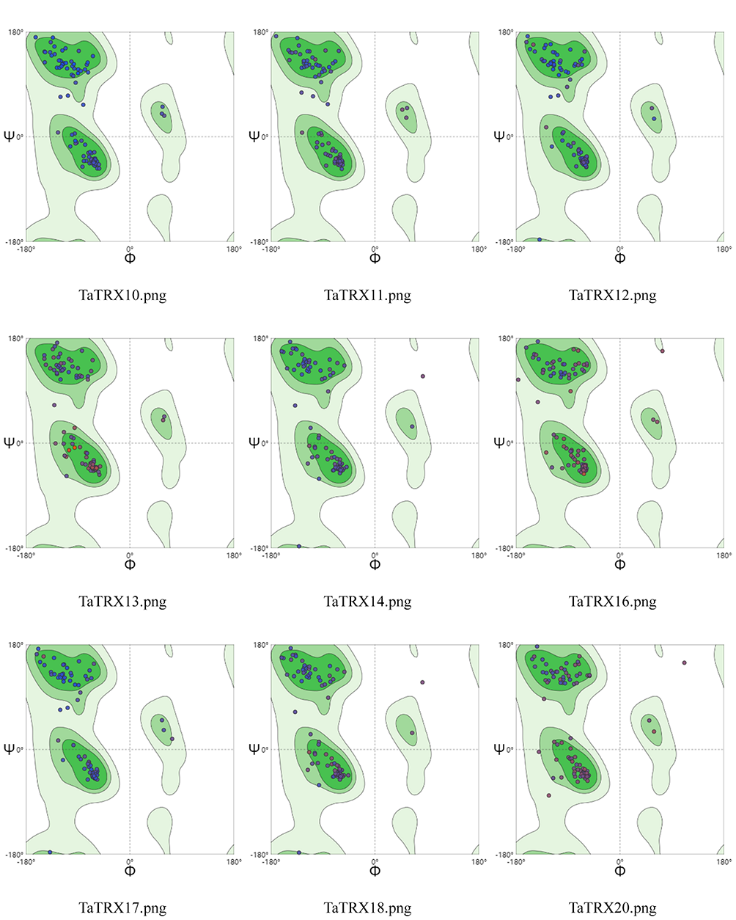


**Figure S4b**. Calculated ramachandran plots for modeled 3D structures of typical TaTRX proteins (TaTRX10-TaTRX20).


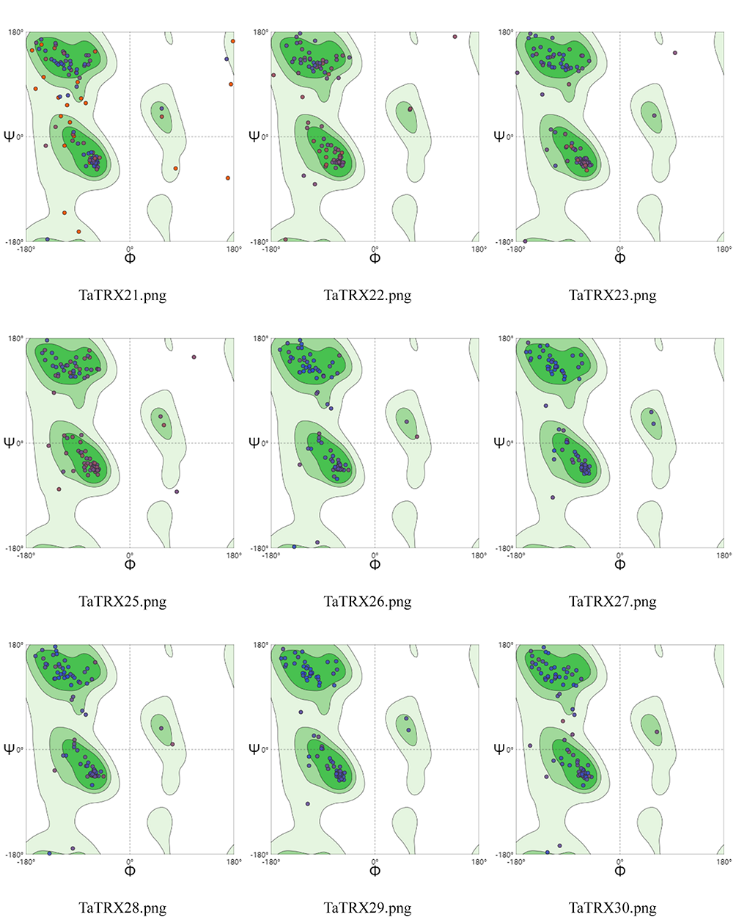


**Figure S4c**. Calculated ramachandran plots for modeled 3D structures of typical TaTRX proteins (TaTRX21-TaTRX30).


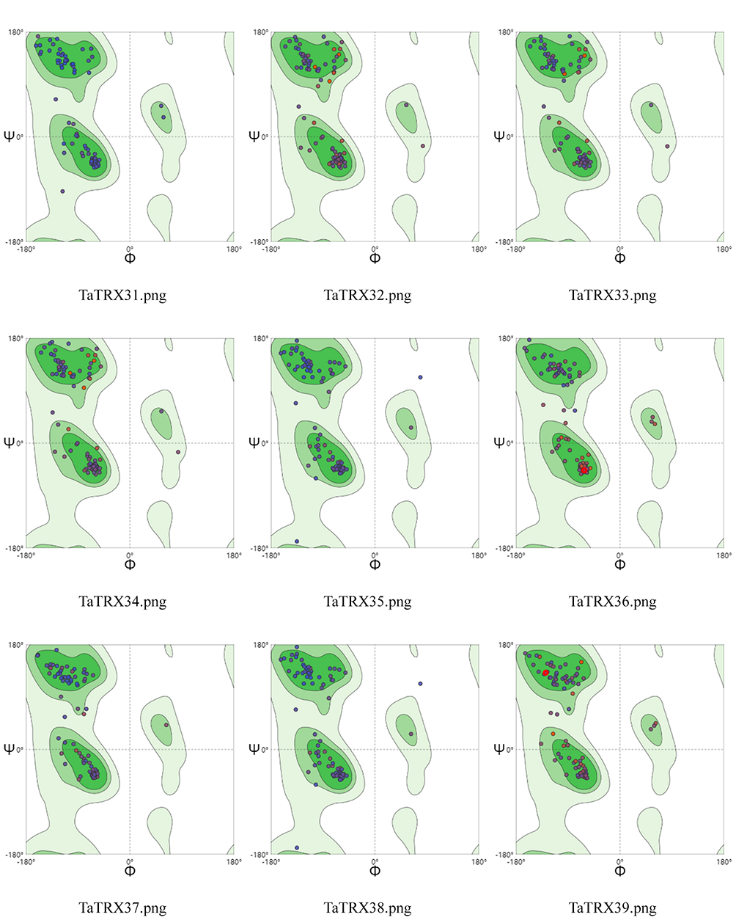


**Figure S4d**. Calculated ramachandran plots for modeled 3D structures of typical TaTRX proteins (TaTRX31-TaTRX39).


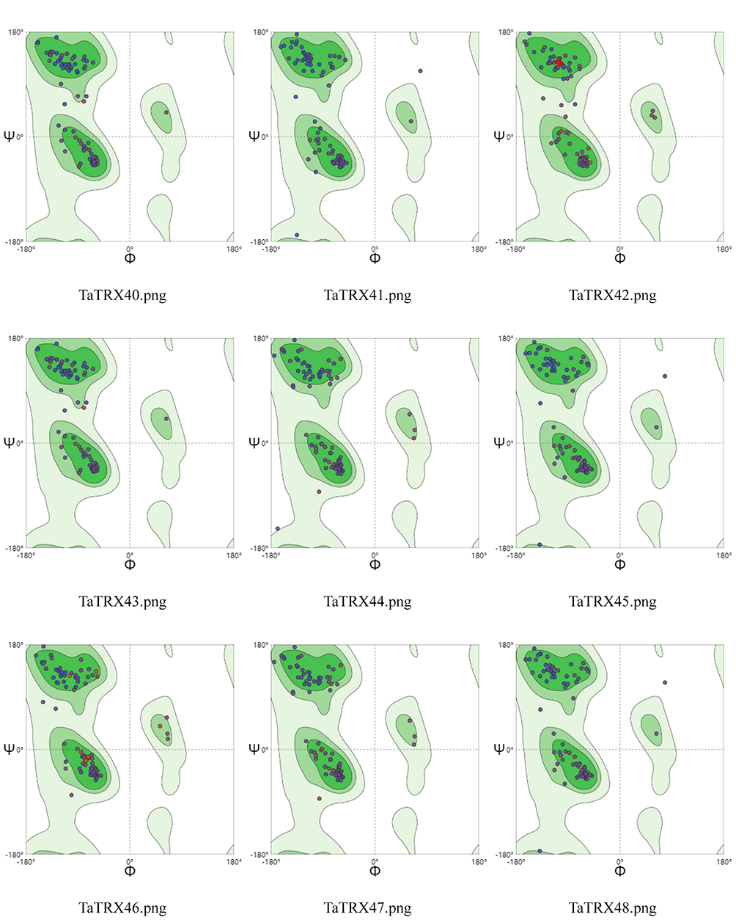


**Figure S4e**. Calculated ramachandran plots for modeled 3D structures of typical TaTRX proteins (TaTRX40-TaTRX48).


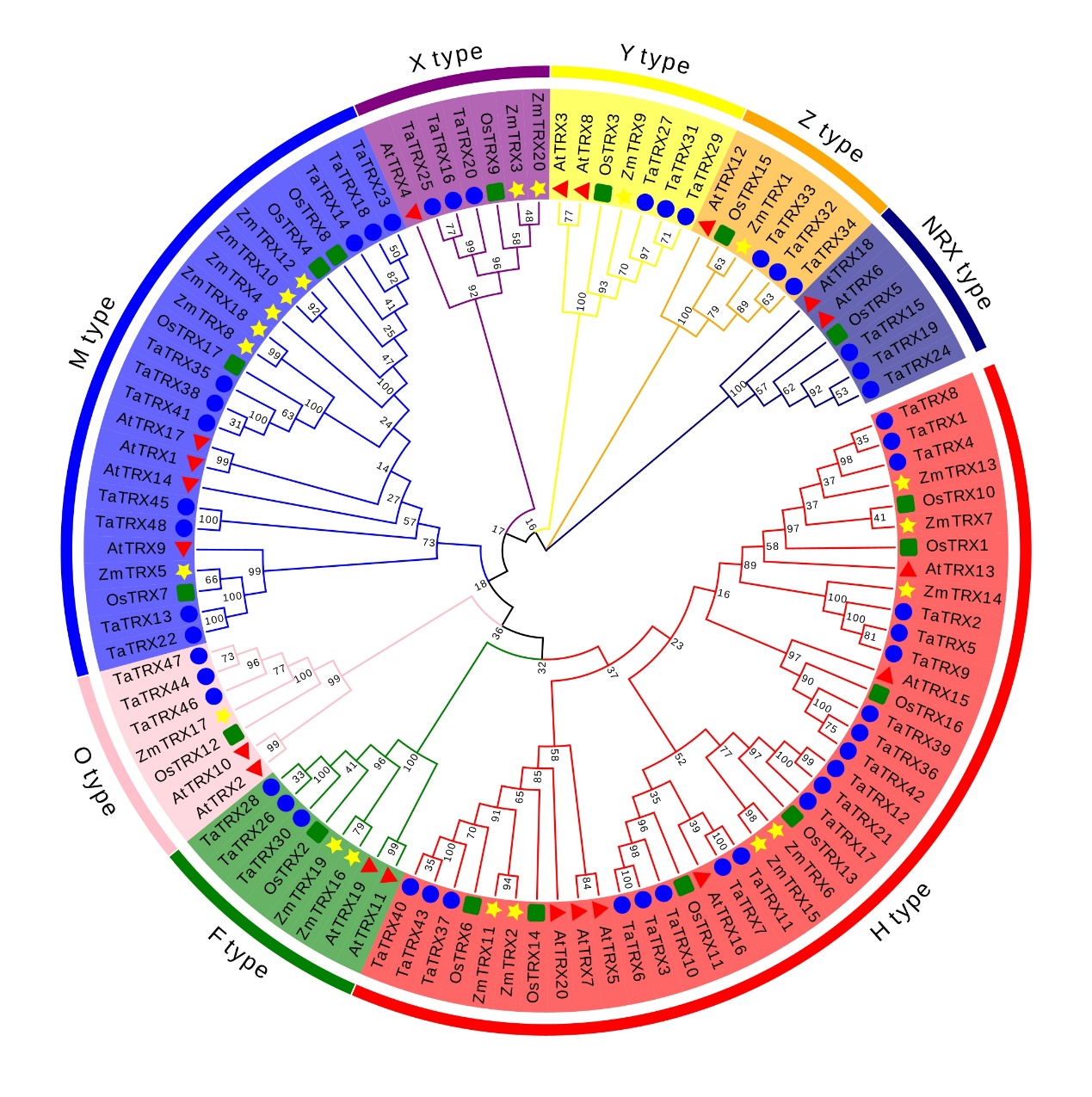


**Figure S5**. Phylogenetic tree of typical TRX proteins using the neighbor-joining method. The 8 different groups were indicated by different colors. The proteins of wheat (*Triticum aestivum* L.; Ta), rice (*Oryza sativa* L.; Os), Arabidopsis (*Arabidopsis thaliana* L.; At), and maize (*Zea mays* L.; Zm) were indicated by different shapes.


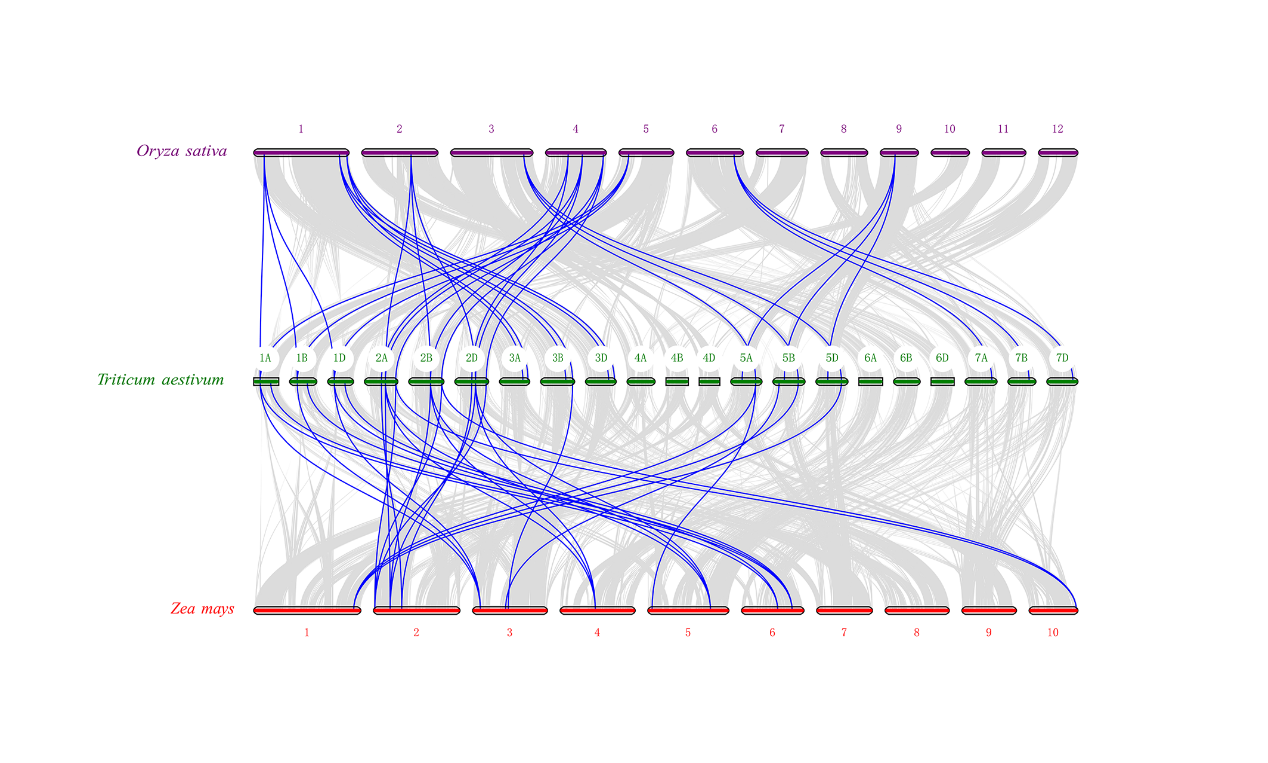


**Figure S6**. Collinearity analyses of typical *TRX* genes between wheat, rice, and maize. Gray lines indicated all collinearity blocks within wheat and other crop genomes, and the duplicated *TRX* gene pairs were highlighted by blue lines. The Arabic numerals in the figure represented the chromosome numbers of the genomes of the above three crops.


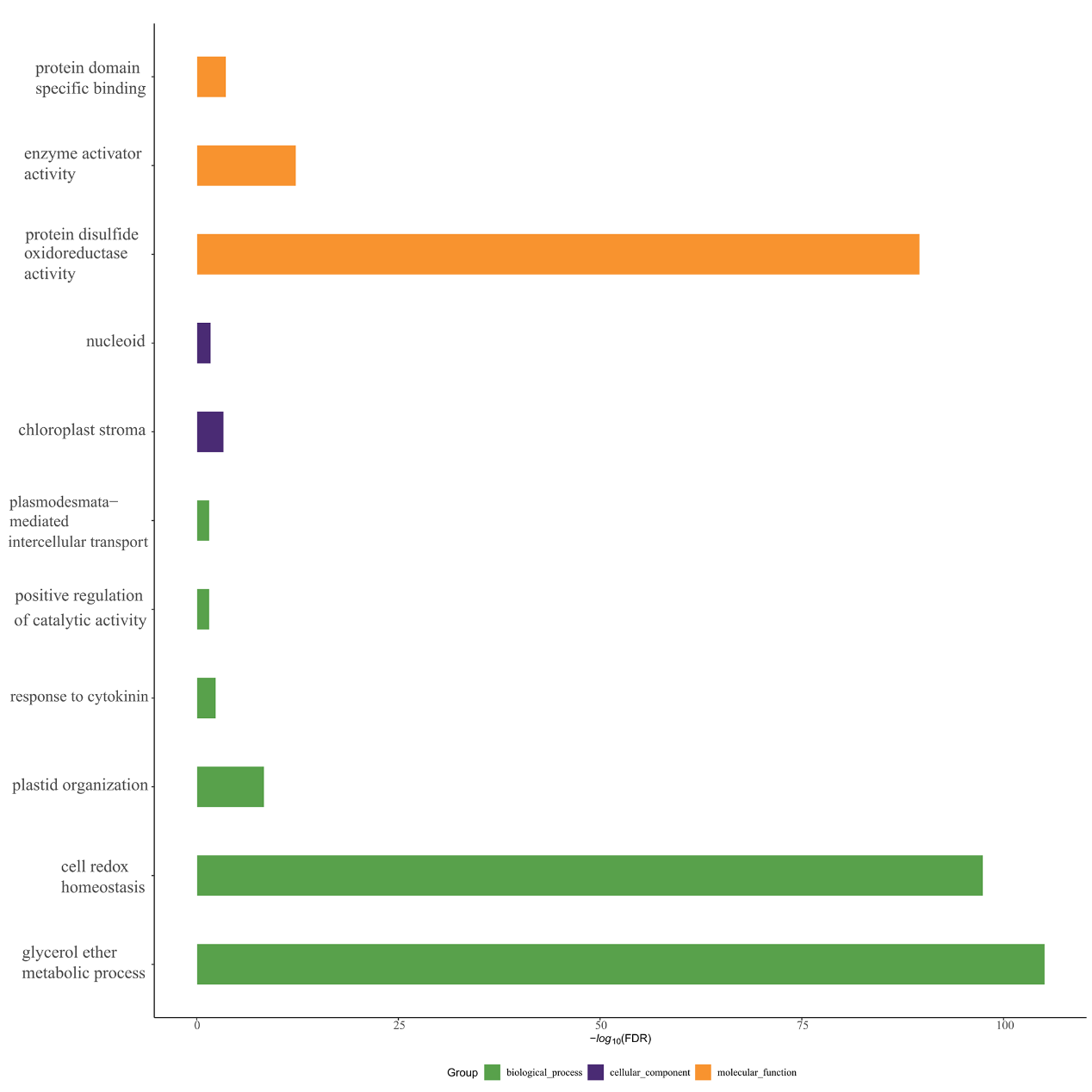


**Figure S7**. Gene Ontology (GO) enrichment analyses of the typical *TaTRX* genes. The green, purple, and orange columns represented the biological process (BP), molecular function (MF), and cellular component (CC) terms, respectively.


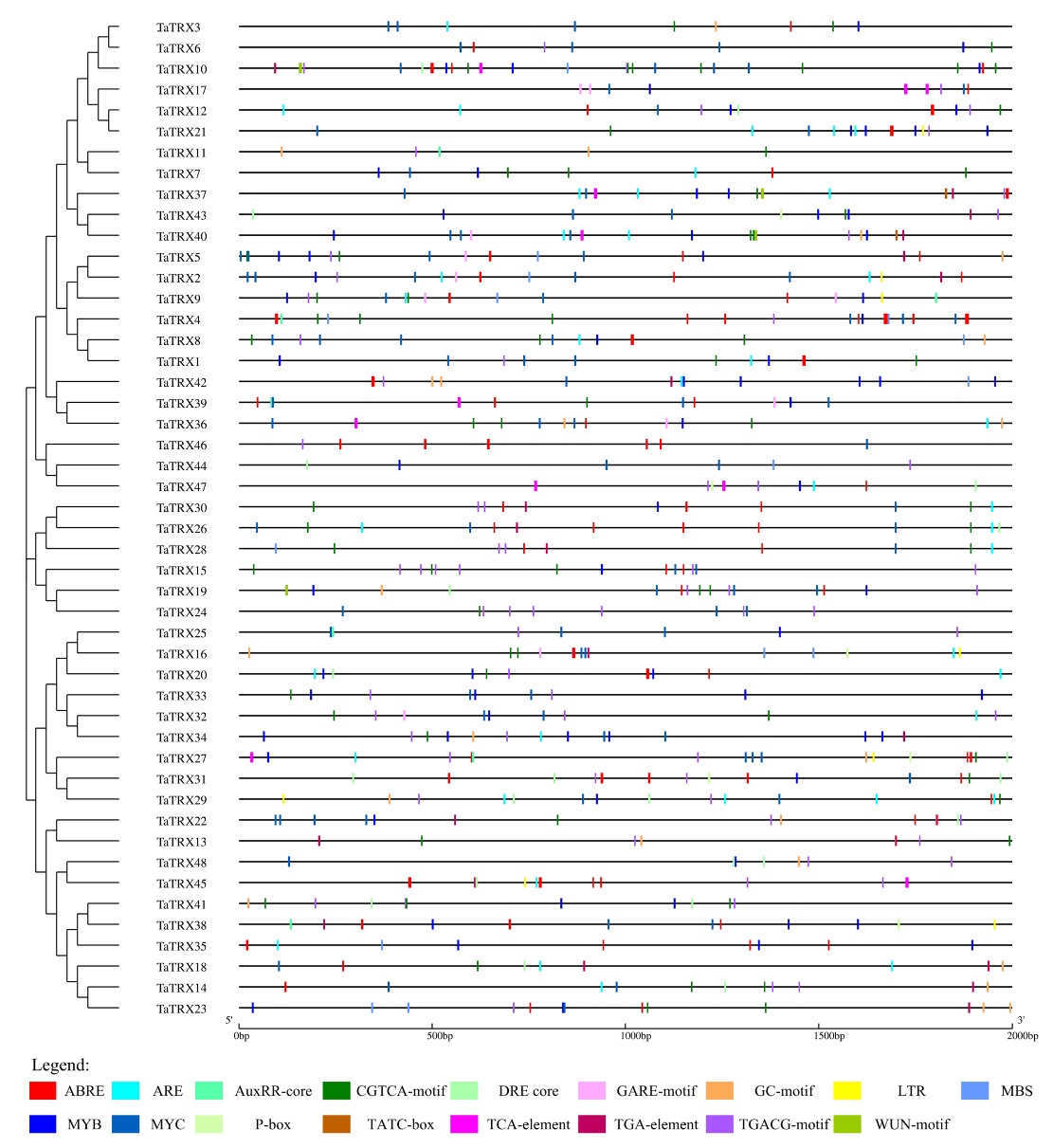


**Figure S8**. *Cis*-elements analysis of promoter sequences of typical *TaTRXs*. Different motifs were indicated by different color rectangles.


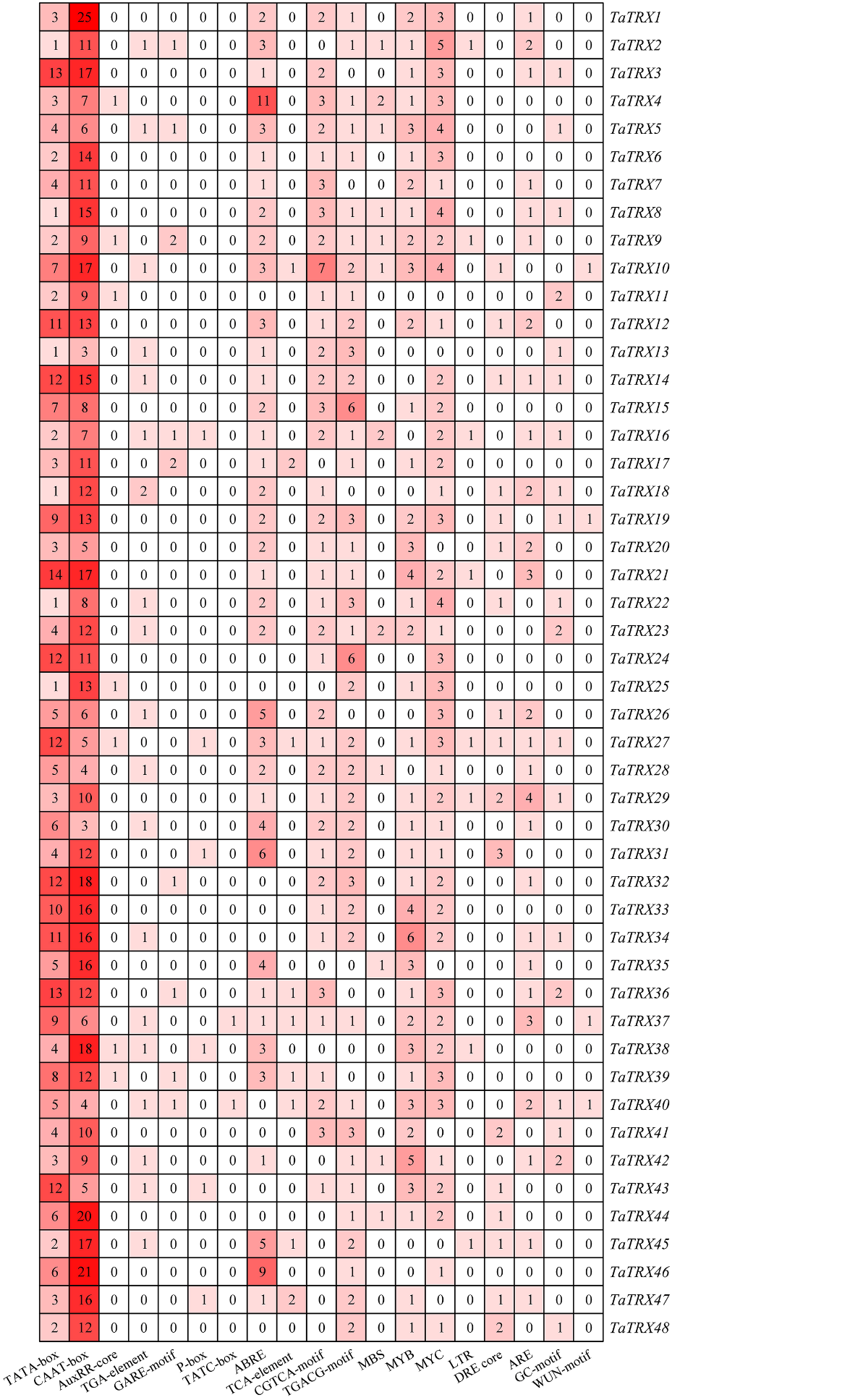


**Figure S9**. Main *cis*-elements in typical *TaTRX* gene promoters. The number in the box means the amount of *cis*-elements.


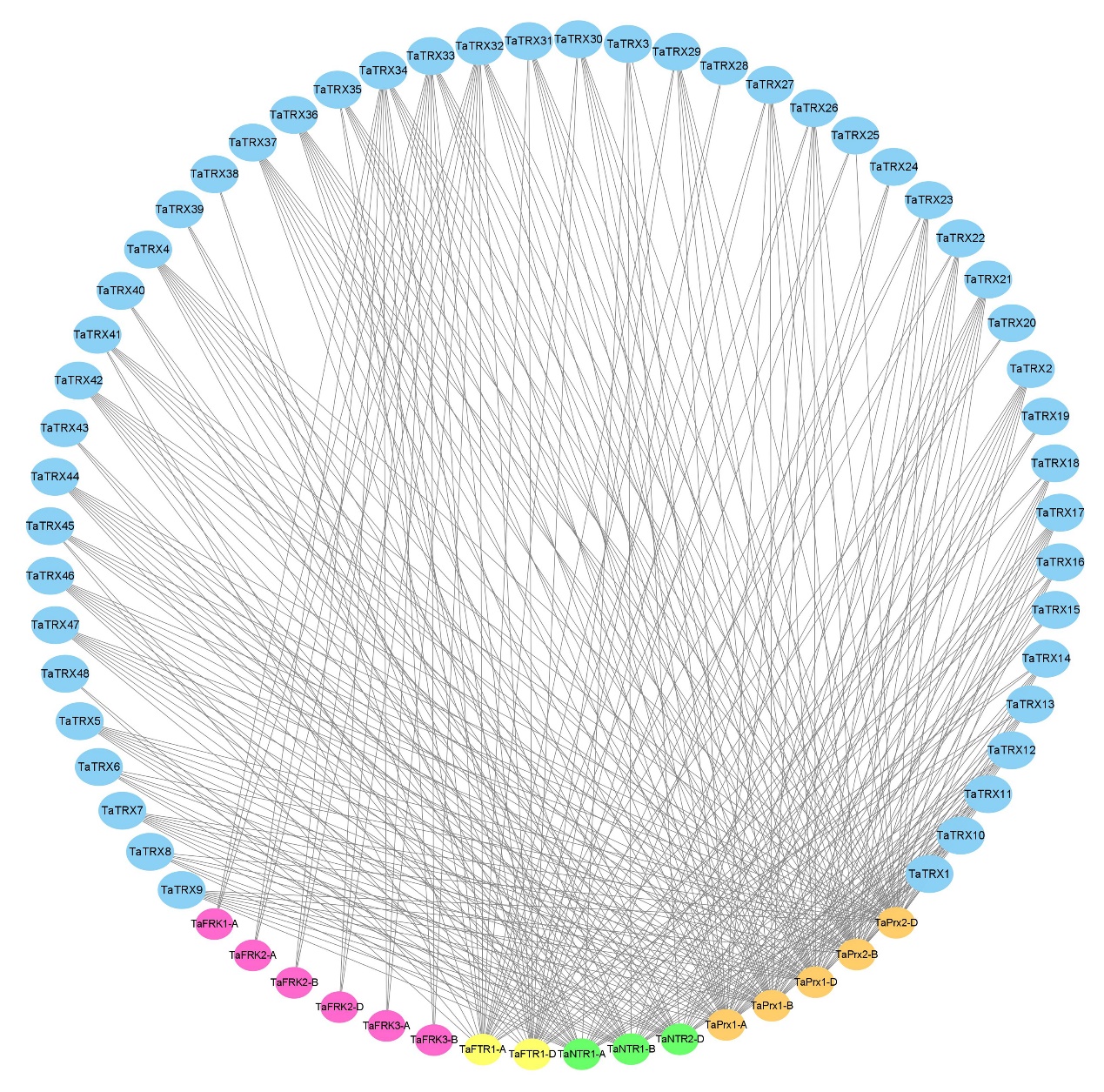


**Figure S10**. Predicted protein interaction networks of typical TaTRX proteins with other wheat proteins using STRING tool. The blue circles, red circles, yellow circles, green circles, and orange circles represented typical TaTRX proteins, TaFRK proteins, TaFTR proteins, TaNTR proteins, and TaPrx proteins respectively. The two circles connected by the gray line represented the interaction between the proteins.
